# The effectiveness of AgNPs synthesized using *Aspergillus floccosus* for controlling *Phoma herbarum* and *Aphis Pomi* de Geer

**DOI:** 10.1007/s11274-026-04855-8

**Published:** 2026-04-18

**Authors:** Onur Aker, İdris Bektaş, Ferit Can Yazdıç

**Affiliations:** 1 Department of Plant and Animal Production, Suluova Vocational School, Amasya University, Amasya, Turkey; 2 Department of Biotechnology, Institue of Graduate Studies in Science, Munzur University, Tunceli, Turkey

**Keywords:** Green synthesis, Ag nanoproduct, Agricultural pest and disease control, Crop health, Apple

## Abstract

This study was conducted to synthesise silver nanoparticles (AgNPs) using *Aspergillus floccosus* fungal extract and to evaluate the pesticidal efficacy of the resulting nanopesticide product against two harmful organisms (pathogenic fungus and pest) affecting apples. The synthesised AgNPs were subjected to a series of characterisation tests (UV-Vis, FTIR, XRD, SEM, TEM, EDX, Zeta Potential) and, based on the results obtained, the AgNPs synthesis was successfully completed. Within the scope of this study, the first registration process has been carried out for the fungal pathogen (*Phoma herbarum*) that causes rot disease in apple fruits. The fungicidal efficacy of five concentrations of AgNPs (10, 25, 50, 100, and 200 mg L⁻¹) was evaluated against this pathogen under both in vitro and in vivo conditions. The results showed that AgNPs exhibited a significant inhibition on the mycelial growth of *Phoma herbarum in vitro*. The 200 mg L⁻¹ concentration showed the highest inhibition percentage (81.5%). Concerning in vivo test, the effect of AgNPs on disease severity index of *Phoma herbarum* in apple fruits was evaluated. The result of this part showed that the concentration 200 mg L⁻¹ of AgNPs exhibits highest fungicidal efficacy disease reduction rates (100%), Following by 67% for AgNPs 100 mg L⁻¹. The insecticidal effects of AgNPs on fourth-stage nymphs and adults of the pest (*Aphis pomi*) were evaluated in laboratory and field applications. For this purpose, AgNPs prepared at five different concentrations (30, 60, 90, 120, and 150 mg L⁻¹) were sprayed on the pests. The results showed that the insecticidal efficacy of AgNPs against *Aphis pomi* was directly related to both the dose and the exposure time. Specifically, the lethal concentration required to kill adults (LC50: 48.9 mg L⁻¹ for laboratory application and 68.4 mg L⁻¹ for orchard application) was higher than the dose required for nymphs (LC50: 32.3 mg L⁻¹ for laboratory and 52.1 mg L⁻¹ for orchard application). A similar trend was observed for lethal time, with adults exhibiting longer required exposure times (LT50: 51.2 h in the lab and 58.4 h in the orchard) compared to nymphs (LT50: 42.0 h in the lab and 45.1 h in the orchard). According to the results obtained, AgNPs synthesised using *Aspergillus floccosus* fungus have been proven to be highly effective at low doses against two major harmful organisms that cause qualitative and quantitative losses in apples. In conclusion, it has been proven that this silver-based nano pesticide product can be used as an alternative and innovative product in combating factors that cause agricultural damage to protect apple health.

**Graphical Abstract:**

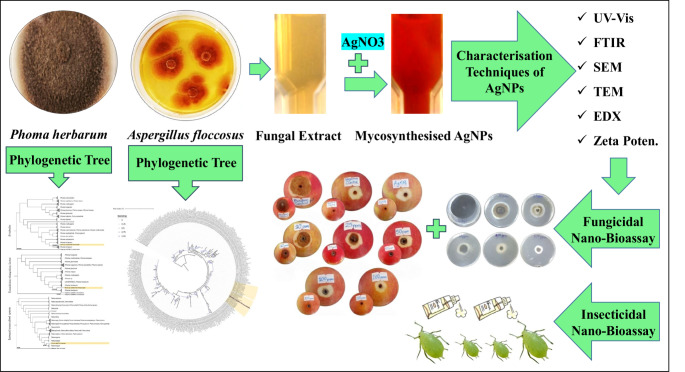

## Introduction

Apple (*Malus domestica* Borkh), which is one of the fruits that people find different flavours and many vitamins together, is a very valuable fruit for human health thanks to the many phytochemical compounds it contains (Jaglan et al. 2022). Thanks to these phytochemical compounds, apple plays an important role for many mechanisms in human cells and is reported to show important effects for the treatment of many important diseases (Hyson 2011). Due to these important features, apple has reached a production capacity of 97.4 million tonnes in an agricultural area of approximately 4.6 million hectares according to the latest agricultural data (Faostat 2025). Apple cultivation suffers significant economic losses every year due to numerous harmful organisms (insects and fungi) (Moinina et al. 2019).

One of these harmful organisms found on apples is the apple aphid (*Aphis pomi* de Geer, 1773, Hemiptera: Aphididae). This pest slows tree growth in young apple trees, causing leaves to curl and fruit discolouration (Blommers 1994; Van Emden and Harrington 2007; Erdogan et al. 2023). It is also a vector of many phytopathogenic viruses and causes mould growth on leaves and fruit due to the honeydew they secrete (Beers et al. 2003). Synthetic chemicals are widely used in production areas to combat this harmful organism. However, due to this harmful insect’s high growth rate and ability to produce multiple generations, its capacity to develop resistance to many synthetic chemicals is very high (Tamaš et al. 2015; Paula et al. 2020; Wojciechowicz-Żytko and Wilk 2023). In addition, the excessive use of synthetic chemicals with different properties causes many different negative effects on public and environmental health (Carvalho 2017; Kim et al. 2017).

Fungal pathogens, like harmful insects, cause serious problems in apple cultivation. Pathogenic fungi significantly affect apple fruit in terms of both quality and quantity, causing irreversible damage (Patriarca 2019). For this reason, producers are conducting intensive chemical control against these pathogenic fungi. However, as a result of excessive chemical control, serious problems have been observed in terms of environmental toxicological risks, pathogen resistance, and public health (Gupta and Gupta 2025). Furthermore, pathogenic fungi isolated from agricultural products each year are diagnosed at the molecular level and added to the literature. This study also represents the first recorded instance in the literature of the pathogenic fungal species (*Phoma herbarum*) isolated from the apple fruit. This pathogenic fungus, diagnosed at the molecular level, causes serious rot disease in apple fruits. Research has shown that if this fungus is not combated, it could cause significant losses in both the quality and quantity of apple fruit within a very short period of time.

Therefore, it is crucial to control this harmful insect and fungal disease, which causes serious problems in apple cultivation. However, priority should be given to the environment and public health when conducting agricultural control measures. Furthermore, it is crucial that the control agent used for agricultural purposes does not cause resistance to develop in organisms that harm apples. For these two important reasons, nanotechnological approaches have come to the fore in agricultural production in recent years (Akhtar et al. 2020; Mondal et al. 2020). Nanotechnological approaches have proven to be not only highly efficient and capable of producing high-quality products, but also extremely beneficial for the environment and public health (Chhipa 2019; Fincheira et al. 2020; Taran et al. 2021; Humbal and Pathak 2023). Nanoparticles, one of the approaches of nanotechnology, act like a pesticide in nano structure thanks to their different properties. Thanks to these features, they have proven that they can protect agricultural products in terms of quality and quantity against factors that cause damage in agricultural production (Deka et al. 2021; Shangguan et al. 2024).

Silver nanoparticles (AgNPs) with metallic properties are frequently used in these pesticide efficacy studies (Rikta and Rajiv 2021; Li et al. 2023). Silver nanoparticles, which are preferred in pesticide efficacy studies, are synthesized using extracts obtained from various organisms (plants, fungi, bacteria, algae, etc.) through a process called green synthesis (Bihal et al. 2023; Ali et al. 2024; Qamar et al. 2024). Silver nanoparticles obtained by green synthesis are frequently preferred by researchers due to their less toxicity, smaller nano size and easier applicability (Mohammadi and Amini 2024). In studies of AgNPs synthesized using fungi, these nano products have been found to be highly effective against many fungal agents and harmful insects that damage agricultural production (Khatua and Ghosh 2025; Panda et al. 2025).

Due to their nano-scale dimensions, these synthesized AgNPs create different effects on the targeted insect or fungus. AgNPs first adhere to the target insect’s cuticle, then penetrate the cuticle layer and enter the insect’s body (Benelli 2018; Shahzad and Manzoor 2021; Awad et al. 2022). AgNPs entering the body are easily absorbed by the digestive system and disrupt many physiological processes occurring at the cellular level, ultimately leading to cell death (Suresh et al. 2018; Awad et al. 2022). One of the most important aspects of this situation is that the target insect has no time to defend itself and therefore cannot develop any resistance mechanism and dies quickly (Benelli 2018; Amjad et al. 2022; Hasoon et al. 2023). It has been reported that synthesized AgNPs cause serious damage to the cell wall and membrane, particularly in fungi (Pal et al. 2007). The damage caused by AgNPs by disrupting cell walls and membranes subsequently leads to the breakdown of intracellular proteins. In the subsequent process, they cause a rapid loss of cellular activity due to an increase in reactive oxygen species (Durán et al. 2016; Al-Otibi et al. 2022).

Studies conducted on synthesized AgNPs have shown that these synthesized nano products are highly effective as nano pesticides and can be used as an alternative to synthetic pesticides. The primary objective of this study is to synthesize AgNPs via green synthesis using the extract of *Aspergillus floccosus* fungus. The second objective of the study is to test the potential use of the synthesized nano products as nano pesticides against two harmful organisms (*P. herbarum* and *A. pomi*) that can cause serious damage to apples. The data obtained from this study will significantly contribute to effective agricultural control measures for apple production. At the same time, the data obtained from this study may inspire researchers to protect the health of different orchards experiencing similar problems. In summary, this study will add a new alternative product to the tools that can be used to combat various harmful factors (insects and fungi) that cause qualitative and quantitative losses in agricultural production.

## Materials and methods

### The fungal strain used in the mycosynthesis process (Morphological-molecular identification and phylogenetic analysis)

Thirty-five rhizosphere soil samples were collected from a depth of 15–20 cm from the rhizosphere of different plants cultivated (onion, wheat, sugar beet, and maize) in agricultural fields in the Suluova region (Amasya, Türkiye, GPS; Latitude: 40.829365/Longitude: 35.648336) for fungal diagnosis (September 2023). Each soil sample brought to the microbiology laboratory in polyethylene bags was first placed in 250 ml Erlenmeyer flasks. Subsequently, 10 g of the relevant soil sample were taken into each Erlenmeyer flask and 90 ml of sterile 0.85% NaCl was added to each flask to bring the samples into suspension. The resulting suspensions were shaken in an orbital shaker at 200 rpm for 20 min and then diluted (10⁻¹ to 10⁻⁹). And then, 200 µl was taken from each diluted suspension and spread onto potato dextrose agar (PDA, Merck, Germany) supplemented with 100 µg/ml streptomycin sulphate. The prepared Petri dishes were incubated at 28 °C for 48 h, and this inoculation and incubation process was repeated a second time to increase the purity of the culture. At the end of the incubation period, the fungal isolates developed in the culture medium were evaluated according to their colony morphology. The *Aspergillus* type isolate was randomly selected according to the method used by Zhang et al. (2013), purified from a single colony, and stored at 4 °C for use in subsequent studies.

During the morphological characterisation of the *Aspergillus* type isolate, its macroscopic characteristics (colour, texture and appearance) and microscopic characteristics (based on conidiophores and conidium structures) were taken into account. These identification procedures were performed under a microscope (Olympus Corporation, light microscope BX53, Tokyo, Japan) and the identification keys established by Samson et al. (2014) were used as a reference. Molecular diagnostic studies have also been conducted to definitively confirm the morphological diagnosis of *Aspergillus* type. DNA isolation was performed from fungal mycelia obtained from fungal isolates grown for 5–8 days in prepared PDA culture medium. After the fungal mycelia were collected into 2 ml Eppendorf tubes, they were disrupted using a homogeniser and DNA extraction was performed using a genomic DNA isolation kit (DNeasy Plant Mini Kit, Qiagen Inc., Valencia, CA). PCR amplification of the ITS region of the ribosomal DNA of the fungal isolate was performed using the universal fungal primers ITS-1 (5′- TCCGTAGGTGAACCTGCG-3′) and ITS-4 (5′- TCCTCCGCTTATTGATATGC-3′) (White et al. 1990). The polymerase chain reaction (PCR) procedure, sequencing process, and phylogenetic analyses were performed according to the method used by Bektaş et al. (2024). The PCR result obtained with the primer was subjected to sequence analysis, and the sequence obtained was compared with the National Centre for Biotechnology Information (NCBI) data using the BLAST (http://www.ncbi.nlm.njh.gov/blast) programme. The prepared sequence series was submitted to the NCBI GenBank database to obtain an accession number (Tamura et al. 2021). Based on the collected data, ITS reference sequences were aligned using Clustal X version 2.1 with the help of utility programmes. Maximum likelihood (ML) phylogenies were constructed using MEGA version X (Kumar et al. 2018a). The Interactive Tree of Life (iTOL) server (https://itol.embl.de/) was used to visualise the obtained phylogenetic trees in greater detail (the *Talaromyces flavus* CBS 310.38 (Ac. No. NR147413.1) sequence was used as an outgroup).

### The mycosynthesis process of the AgNPs

Mycosynthesis of *Aspergillus floccosus* (GenBank of NCBI accession number: PQ849182.1) was performed as described by Skanda et al. (2022) with some modifications. The *A. floccosus* isolate was incubated on potato dextrose agar (PDA) at 28 ± 2 °C for seven days. In the next step, five mycelium discs (Ø 10 mm) taken from the PDA medium (using a fungal punch) were inoculated into a medium containing 250 ml of potato dextrose broth (PDB). Subsequently, this mixture was incubated at 28 ± 2 °C for 10 days at 120 rpm in an orbital shaker. After the inoculated medium was filtered with filter paper (Whatman No. 1) to collect the fungal biomass.The filtrate was washed three times with sterile distilled water to make it more pure. Forty g of fungal biomass was mixed with 400 mL of distilled distilled water and incubated at room temperature with shaking at 120 rpm for 72 h. Three days later, this mixture was centrifuged at 5000 rpm for 20 min and 400 mL of this fungal biomass filtrate was slowly reacted with 1 mM AgNO_3_ (400 mL). After observing that the colour of this fungal biomass filtrate changed from very light yellow to orangish brown, it was left to incubate for ten days at room temperature in a dark environment for the maturation process of silver nanoparticles. The silver nanoparticle residue obtained at the end of ten days was subjected to distilled pure water and 5000 rpm centrifugation three times. These processes were carried out three times, after the washing process was thoroughly completed, it was left to dry in a 60 °C drying oven for 48 h and then the nanoparticle powders obtained for characterisation tests were taken into sample tubes.

### Characterisation identification processes of the AgNPs

A series of procedures are required to determine the characterisation of the nanoparticle obtained at the end of the nano-synthesis process. These procedures are; UV-Vis absorption (UV 1601-Shimadzu) to obtain UV-Vis spectrum graph, FTIR spectroscopy (Jasco 430 model Spectrometer) to create FTIR spectrum graph, XRD diffractometer (Rigaku SmartLab 9 kW diffractometer, Cu Kα (λ = 1, 54056 Å), Scanning Electron Microscopy (SEM), Transmission Electron Microscopy (TEM), Energy-Dispersive X-ray Spectroscopy (EDX) (JEOL JSM-7001 F for SEM, TEM and EDX - Japan) and Zeta potential (Nano-ZS90 System, Malvern Inc. Malvern Inc., Malvern, UK) and Zeta potential (Nano-ZS90 System, Malvern Inc., Malvern, UK) analyses (Skanda et al. 2022).

### The pathogenic fungal strain (Morphological-molecular identification and phylogenetic analysis)

In the districts of Suluova and Merzifon (Amasya, Türkiye), diseased samples were collected from 35 apple trees showing symptoms of disease (October 2023). The collected apple samples were brought to the laboratory and stored at 4 °C until pathogenic fungi were isolated. The infected apples were thoroughly washed with sterile water and cut aseptically into 2 to 3 mm pieces. The small pieces were then carefully placed on PDA plates containing chloramphenicol to prevent bacterial growth. The prepared plates were incubated for 5–7 days at 28 ± 2 °C under continuous light to promote fungal growth. After the incubation period was complete, the resulting fungal colonies were carefully examined and classified according to morphological characteristics such as colour, texture and growth pattern. Representative isolates were subcultured to ensure purity and to facilitate further characterisation and pathogenicity testing (Kurt et al. 2020). The conidial structures of the fungi were analysed under a light microscope (Olympus CX21LED). The morphological characterisation process was carried out using standard literature that provides detailed guidelines for distinguishing the various features and characteristics of fungi (Domsch et al. 1980). To perform the molecular characterisation of the fungal isolate, genomic DNA was extracted from 10-day-old mycelium obtained from a single-spore culture grown on PDA at 28 °C. The extraction of total genomic DNA was performed using the DNeasy Plant Mini Kit (QIAGEN, Hilden, Germany) to ensure optimal yield and purity of the DNA (Uysal et al. 2022). To assist in the molecular characterisation of the fungal isolate, three genomic loci, ITS4/ITS5 (White et al. 1990), 1-α (tef1) (Carbone and Kohn 1999), and β-tubulin (tub2) (Glass and Donaldson 1995), were amplified and sequenced. The PCR amplification mixture and protocols were applied as described in the literature (Bezerra et al. 2021; Aiello et al. 2022). First, each sequence was analysed for potential anomalies or errors, and then alignments were performed to verify the identity of the fungal isolates. The sequences obtained from PCR amplification were compared with a public database using the Basic Local Alignment Search Tool (BLAST) (http://www.ncbi.nlm.nih.gov/BLAST) and selected as the closest phylogenetic neighbour (Hentschel et al. 2001). These sequences were subsequently aligned using the Clustal X v.2.1 programme, enabling the identification of homologous regions between different fungal isolates (Larkin et al. 2007).

In the construction of phylogenetic dendrograms, the internal transcribed spacer (ITS), translation elongation factor 1-α (tef1) and β-tubulin (tub2) 16 S rDNA gene sequences were constructed using the Neighbourhood Joining (NJ), Maximum Likelihood (ML), and Maximum Parsimony (MP) methods, employing the MEGA 11 software and Tamura-Nei models. A p-distance matrix for nucleotides was utilized, with the pairwise gap deletion option selected. The analysis includes 1000 bootstrap replications to ensure the robustness and reliability of the phylogenetic relationships shown in the dendrograms (Tamura et al. 2021). The resulting phylogenetic trees were converted into Newick format, which efficiently represents the evolutionary relationships between fungal isolates. The Interactive Tree of Life (iTOL) server was used to provide a more detailed visualisation of these phylogenetic trees (https://itol.embl.de/). This platform provides tools for the interactive analysis and customisation of displayed phylogenetic data, thereby facilitating a better understanding of the relationships between the species under investigation (Letunic and Bork 2021). Finally, the sequences obtained from the amplification of the ITS, 1-α (tef1) and β-tubulin (tub2) regions were deposited in GenBank.

### The pathogenicity test of the pathogenic fungal strain

Apple fruits (local Amasya cultivar) were selected to test the pathogenicity of the pathogenic fungal isolate subjected to diagnostic procedures. Apple orchards that had not been exposed to pesticides in any way (in Amasya province) were identified, and tests were conducted on healthy apple fruits collected from these orchards using the wound/drop method adapted by Aiello et al. (2015) and Guarnaccia et al. (2017). First, a spore suspension with a concentration of 1 × 10⁶ conidia mL⁻¹ was prepared from cultures of the pathogenic fungal isolate developed in PDA medium at 28 °C for 10 days. For this purpose, the conidial suspension was first prepared in sterilized distilled water + 0.05% (v/v) (Tween^®^ 80). The conidial suspension was then adjusted to a level of 1 × 10⁶ conidia mL^− 1^ using serial dilutions with a hemocytometer. Apples were prepared for this test, and 100 µL of the prepared spore suspension was injected into each inoculation point on each fruit. Ten apples were prepared for each treatment for the pathogenicity test. These procedures were repeated three times throughout the pathogenicity test. The apples in the control group were treated only with pure water. In the assessment of fungal disease, the percentage of infected inoculation points for the pathogenic fungal isolate was calculated based on disease assessments conducted 10 days after inoculation (28 ± 2 ℃, 70 ± 5% RH and 12:12 h L/D photoperiod) (Tekiner et al. 2020). It was compared with the fungus inoculated by re-isolating it from apple tissues showing signs of decay.

$$\:\mathrm{P}\mathrm{e}\mathrm{r}\mathrm{c}\mathrm{e}\mathrm{n}\mathrm{t}\mathrm{a}\mathrm{g}\mathrm{e}\:\left(\mathrm{\%}\right)=\frac{\mathrm{I}\mathrm{n}\mathrm{f}\mathrm{e}\mathrm{c}\mathrm{t}\mathrm{e}\mathrm{d}\:\mathrm{i}\mathrm{n}\mathrm{o}\mathrm{c}\mathrm{u}\mathrm{l}\mathrm{a}\mathrm{t}\mathrm{i}\mathrm{o}\mathrm{n}\:\mathrm{p}\mathrm{o}\mathrm{i}\mathrm{n}\mathrm{t}\mathrm{s}}{\mathrm{I}\mathrm{n}\mathrm{o}\mathrm{c}\mathrm{u}\mathrm{l}\mathrm{a}\mathrm{t}\mathrm{i}\mathrm{o}\mathrm{n}\:\mathrm{p}\mathrm{o}\mathrm{i}\mathrm{n}\mathrm{t}\mathrm{s}}\times\:100$$

### Fungicidal nano-bioassay process

A series of test procedures were carried out to determine the efficacy of synthesised AgNPs against the apple rot pathogen fungus under in vitro. First, Petri dishes (Ø 90 mm) containing PDA medium were prepared in triplicate for the control (0 mg L⁻¹) and five different AgNPs concentrations (10, 25, 50, 100 and 200 mg L⁻¹). A total of six different AgNPs concentrations were prepared and concentration-dependent naming procedures were applied (AgNP-0, AgNP-10, AgNP-25, AgNP-50, AgNP-100 and AgNP-200). PDA media were prepared for each concentration value (6 different values). The prepared PDA medium was then sterilized by autoclaving at 121 °C for 15 min. Finally, the prepared PDA medium was poured into pre-labeled Petri dishes. In vitro tests were performed in a laboratory setting using standard sterile techniques with three replicates. After all Petri dishes were prepared, they were incubated at 28 ± 2 °C for 24 h. After 24 h of incubation, the pathogenic fungal culture was collected using a special fungal sampling tool (a metal rod with a 10 mm diameter piercing tip) and then superficially inoculated into the centre of all prepared Petri dish media. All Petri dishes were then incubated at 28 ± 2 °C for 10 days. At the end of the incubation period, the diameters of the pathogenic fungal mycelium growing in the Petri dishes were measured using an electronic caliper and recorded. In the evaluation, the moment when the diameter of the mycelium formed in the control Petri dishes reached the edge of the control Petri dishes was considered fixed. The extent to which the mycelial growth of the pathogenic fungus was inhibited in Petri dishes containing different AgNPs concentrations was calculated using the following formula (Kim et al. 2012).

$$\begin{array}{c}Inhibition\:\left(\%\right)\\=\frac{Fungal\:diameter\:in\:control\:petri\:dishes-Fungal\:diameter\:in\:petri\:dishes\:with\:AgNPs\:added}{Fungal\:diameter\:in\:control\:petri\:dishes}\times\:100\end{array}\:$$

For in vivo tests, healthy, undamaged apple fruits of approximately the same size (local Amasya cultivar, Ø 80–90 mm) were selected. The fruits has been harvested from apple orchards (in Amasya province) that have not been exposed to pesticides in any way. The apple fruits were first subjected to surface sterilisation with 70% ethanol for two minutes, then thoroughly washed twice with sterile distilled water, dried, and stored at 4 °C until ready for use. The pathogenic fungal isolate causing apple rot was placed on PDA medium and incubated at 28 °C for seven days. Ten millilitres of sterile distilled water were added to the prepared seven-day pure culture, and the medium was stirred with a sterile rod to obtain a spore suspension. This suspension was filtered using Whatman filter paper to obtain the sport solution. The resulting spore suspension was adjusted to a spore concentration of 1 × 10⁶ conidia mL⁻¹ using a hemocytometer as described above. The in vivo study was designed to include a total of eight treatment groups: one positive control, one negative control, one AgNP effect control group, and five treatments containing AgNP at different concentrations (10, 25, 50, 100, and 200 mg L⁻¹). Each group application was tested using 5 apples in each group. In this way, the entire in vivo test procedure is designed to be repeated five times. Before application, the nanoparticle suspensions were sonicated and vortexed to ensure a homogeneous distribution and to minimize aggregation. For the single-point pathogenicity inoculation procedure in apple fruits, a specially designed fungal inoculation needle (3 mm deep and 3 mm wide) that can be sterilised for each application was used. Using this inoculation rod, lesion points could be carefully created on each fruit by inoculating 100 µL of the desired substance at a single point determined on each fruit. To determine the effectiveness of positive control on the fruits, only 100 µL of a 10⁶ spores/ml suspension was inoculated at the relevant point. To determine the effectiveness of the negative control, only 100 µL of pure water was inoculated onto the relevant point of the prepared fruits. To determine the effect that synthesised AgNPs alone could have on fruit, only 100 µL of AgNP at a concentration of 200 mg L⁻¹ was inoculated onto the relevant part of the fruit. For the remaining five AgNPs applications (AgNP-10, AgNP-25, AgNP-50, AgNP-100 and AgNP-200), apple fruits were inoculated with 100 µL of a 10⁶ spores/ml fungal suspension. These apple fruits were left to incubate for 24 h (28 ± 2 ℃, 70 ± 5% RH and 12:12 h L/D photoperiod). Following the incubation period, injections were administered into the inoculation sites of these apple fruits to determine the in vivo efficacy of AgNPs. For this procedure, 100 µL of AgNP at the relevant concentration (10, 25, 50, 100 and 200 mg L⁻¹) was injected into the relevant inoculation points of each apple fruit prepared for this process according to its group. There were five apples for each application procedure, and the procedures were repeated a total of five times for in vivo testing. After all preparations were completed for the total of eight applications tested, all apple fruits were incubated for ten days (28 ± 2 ℃, 70 ± 5% RH and 12:12 h L/D photoperiod). At the end of the incubation period, disease scores were assessed and the diameters (mm) of lesions developing in the vaccinated areas were measured using an electronic caliper and recorded. The in vivo efficacy of AgNPs suspension against apple rot disease was determined by comparing the recorded measurement values with the control application. In each application, the diameters of the observed fruit rot were classified on a 0–5 point scale, and disease severity indices and disease reduction rates/status were calculated as percentages according to the following formulas (Promwee et al. 2017).

(0: no rot, 1: 0–10 mm, 2: 10–20 mm, 3: 20–30 mm, 4: 30–40 mm, 5: 40–50 mm. radial rot of apple tissue)

$$\:\mathrm{D}\mathrm{S}\mathrm{I}\:\left(\mathrm{\%}\right)=\frac{\varSigma\:\left(S\:x\:A\right)}{\mathrm{M}\:\mathrm{x}\:\mathrm{T}}\times\:100$$

S: scale; A: tuber quantity; M: maximum level; T: total number of apple fruits.

The disease reduction rate (R) was calculated using the Abbott formula (Abbott 1925),

$$\:\:\:\:R\left(\mathrm{\%}\right)=\frac{(\mathrm{A}\:-\:\mathrm{B})}{\mathrm{A}}\times\:100$$

‘A’ disease severity level observed in the positive control group, ‘B’ disease severity level in samples treated with mycosynthesised AgNPs against pathogenic fungi.

Following completion of the in vivo efficacy test, re-isolation was performed using decayed apple fruits, and the pathogenic fungal isolate obtained from this was morphologically compared with the original strain (Recep et al. 2009).

### Insecticidal nano-bioassay process

A large number of second-third instar nymphs of *A. pomi* were carefully collected from pesticide-free apple orchards and brought to the Entomology laboratory where the nymphs were cultured (24 ± 2 ℃, 65 ± 5% RH and 16:8 h L/D photoperiod). A large number of new generation individuals were obtained from this apple pest culture and the new generation of young (48 h old) wingless adults and fourth instar nymphs (48 h old) were used for bioassay studies in the laboratory and apple orchards.

For the bioassay performed in the laboratory, plastic Petri dishes (Ø 60 mm) were used and sterilised and moistened blotting papers (Whatman No. 1, Ø 60 mm disc) were placed on the bottom of these Petri dishes. Fresh apple leaves (cut into Ø 60 mm discs) collected from apple trees and thoroughly cleaned with distilled water were placed in Petri dishes. Ten young wingless adults were carefully placed in each of these Petri dishes using a fine bristle brush. This procedure was established on the basis of randomised experimental plots with ten replications for each of the five different dose treatments (30, 60, 90, 120 and 150 mg L⁻¹). Spraying method was preferred for the bioassay, accordingly, AgNPs suspension prepared at five different doses were sprayed ten cm above the Petri dishes with the help of Nano-Spray Oximeter and then the top lids of these Petri dishes were closed. Distilled water was preferred for spraying the control groups. Bioassay studies of fourth instar nymphs were carried out in the same way as young adults. After the Petri dishes were prepared in ten replicates for each of the five different dose treatments, the Petri dishes were sprayed and only distilled water was sprayed against the nymphs in the Petri dishes selected as the control group. All Petri dishes prepared for the biological experiment of young adults and nymphs were placed in acclimatisation cabinets (24 ± 2 ℃, 65 ± 5% relative humidity and 16:8 h L/D photoperiod) and the Petri dishes were checked at 24 h intervals for four days. Dead adults and nymphs were noted at each daily control period and dead individuals were removed from the Petri dishes.

For the bioassay in apple orchards, treatments were carried out on a 10–12 year old apple tree with healthy leaves in an orchard where chemical pesticides were not used (June 2025, Amasya Province, Turkey). For the bioassay, young leaves of the apple tree were identified and washed thoroughly with distilled water. Ten young adults of *A. pomi* were carefully penetrated into each of the prepared leaves with a fine bristle brush. Ten leaves for each different AgNPs dose (30, 60, 90, 120 and 150 mg L⁻¹) were prepared in this way and then five different AgNPs doses were applied by spraying from a height of ten cm on the upper parts of the leaves prepared separately with the help of Nano-Spray Oximeter. Some of the prepared leaves were separated as a control group and only distilled water was used for spraying the upper parts of these leaves where the pests penetrated. The bioassay studies of fourth instar nymphs in apple orchards were carried out in the same way as the application method designed for young adults. Five different doses of AgNPs and distilled water as control were sprayed on the upper part of these leaves where nymphs penetrated. Each of the prepared and sprayed leaves was wrapped in a very fine mesh cheesecloth and the cheesecloth was tied softly at the stem of the leaves. The selected apple tree in the apple orchard was checked every day for 4 days after the application and dead adults and nymphs on the cheesecloth and leaves were recorded.

### Data analysis

The statistical significance of all results obtained in in vitro tests (measuring the radial diameter of the fungus in Petri dishes) and in vivo tests (measuring the diameter of the rot caused by the pathogenic fungus on apples) was characterised by Duncan’s multiple range test (One-Way ANOVA test, SPSS 2017; version 25.0) (*p* ≤ 0.05). In this nano-insecticidal study against adults and nymphs of *A. pomi*, the Abbott formula was used to correct the mortality observed in the control group (Abbott 1925). The results of dose- and time-dependent mortality rates on adults and nymphs depending on two different application methods of nanoparticles prepared in five different doses were subjected to Regression-Probit (SPSS 2017; Version 25.0) analysis. According to the results of the analysis, lethal concentration values (LC50-LC95) and lethal time values (LT50-LT95) for adults and nymphs of the pest were determined and the results were tabulated.

## Results and discussion

### Characterisation analyses of the mycosynthesised AgNPs

#### UV–VIS

**Fig. 1:**
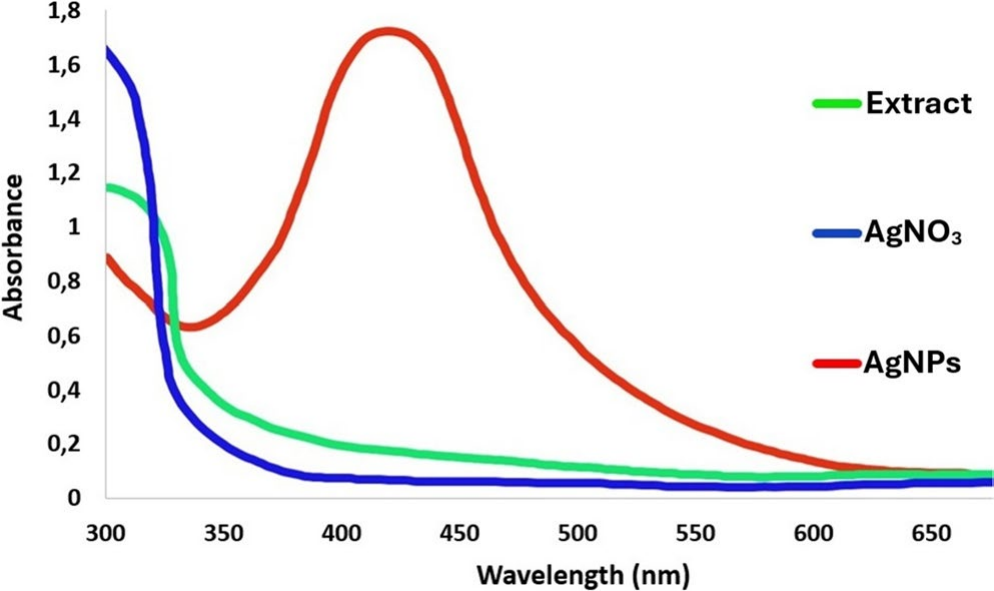
UV–Vis spectrum

Due to the reduction of silver ions due to the redox reaction during the mycosynthesis process, a colour change towards darkening is observed in the suspension prepared in this process and this is mainly due to surface plasmon resonance (SPR) (El-Bendary et al. 2021; Chi et al. 2022). In this mycosynthesis process, in which many secondary compounds of fungal origin act as reducing agents, surface plasmon resonance (SPR) is stimulated by the reduction of silver ions, resulting in both a colour change and a specific peak in the absorbance of the spectrum measured in UV (El‐Bendary et al. 2021; Chi et al. 2022). As a result of the UV spectrum reading of the silver nanoparticle prepared at the end of this mycosynthesis process, a peak value at 424 nm wavelength was recorded (Fig. 1). It was also reported that peaks at 400 nm and 440 nm wavelengths occurred in the spectrum readings obtained at the end of mycosynthesis processes using *Aspergillus* spp. fungus, while no peak value was recorded for the fungal extract (El‐Bendary et al. 2021; Awad et al. 2022; Chi et al. 2022; Rai et al. 2023).

### FTIR

**Fig. 2:**
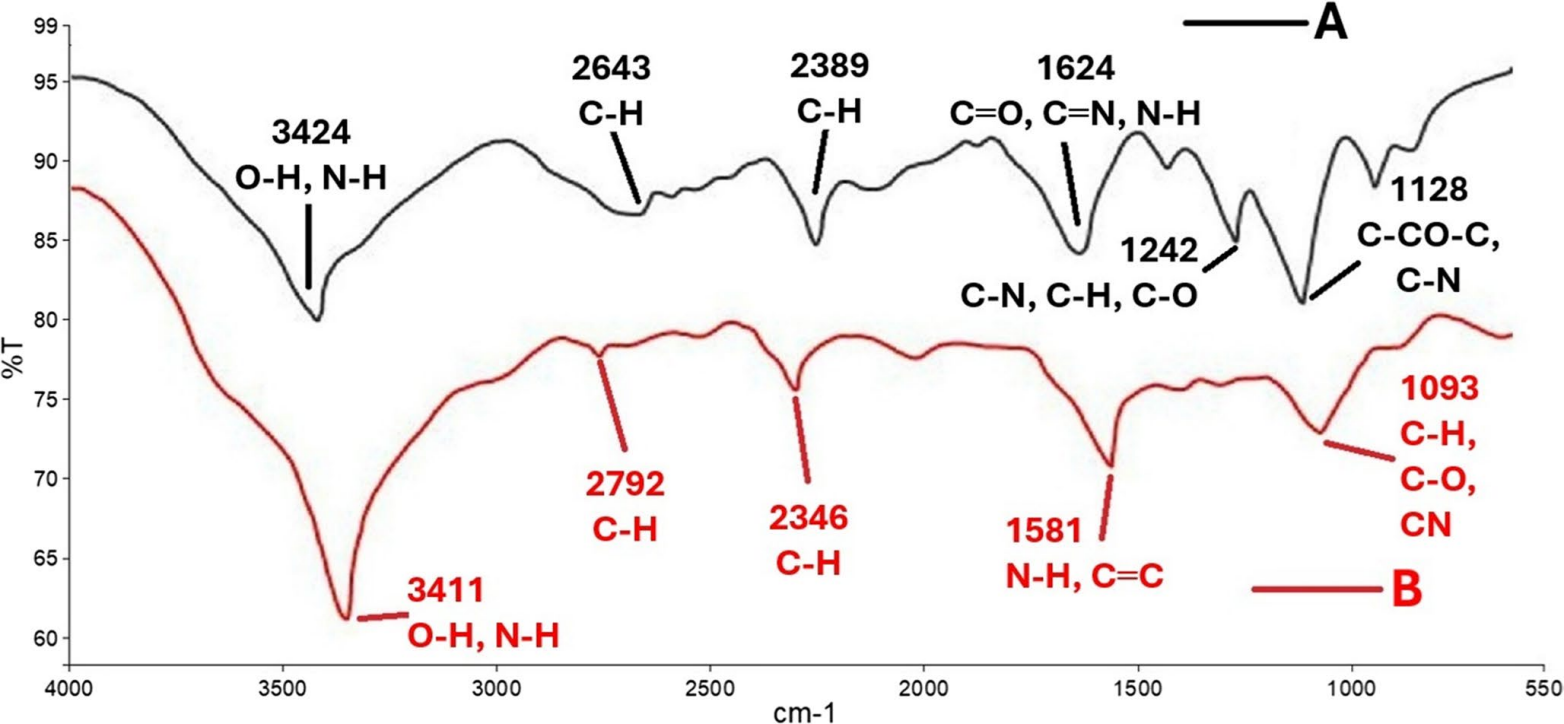
FTIR spectrum of fungal extracts (**A**) and AgNPs (**B**)

It is known that secondary compounds obtained from fungal extract act as reducing and incorporating agents in the synthesis process and mediate the reduction of silver Ag^+^ ions to Ag^0^ ions in this process (El-Bendary et al. 2021; Awad et al. 2022; Chi et al. 2022; Rai et al. 2023). In these reduction processes, it is reported that some functional groups come to the fore depending on the substance and are more effective in the synthesis process (Awad et al. 2022; Chi et al. 2022). The presence of these effective groups (such as alcohols, phenols, alkanes, alkyls, aldehydes, amines etc.) manifests itself in different peak values in the spectrum readings of both the extract and the silver nanoparticle (El‐Bendary et al. 2021; Awad et al. 2022; Chi et al. 2022; Rai et al. 2023). In this study, the functional groups and peak values of the extract and nanoparticle were determined by FTIR spectrum analysis and the similarities and differences between them were shown (Fig. 2). According to the graphical states of the vibrations occurring in different spectrum bands, the bond structures caused by different functional groups were differentiated by the reduction of the silver nanoparticle. In similar studies using *Aspergillus* spp. fungi, it was reported that the functional groups affected by the extract and the synthesised silver nanoparticle were similar to each other and small differences occurred between them (El‐Bendary et al. 2021; Awad et al. 2022; Chi et al. 2022).

### XRD

**Fig. 3:**
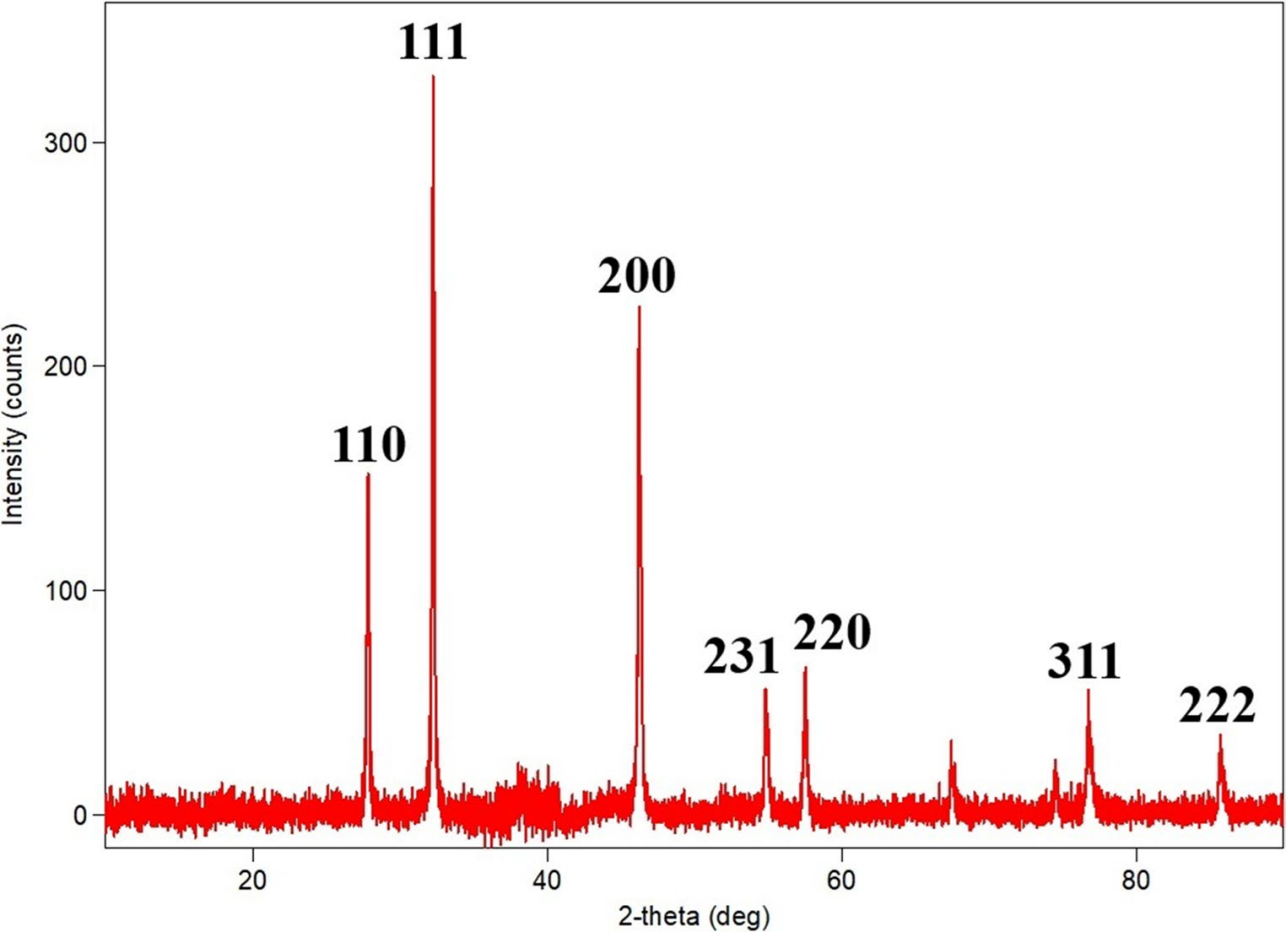
XRD pattern of AgNPs

The diffraction reflections of the synthesised silver nanoparticles were determined by XRD analysis (between 10° and 90° at 2θ angle values) and nine reflection values were obtained accordingly. Seven of these diffraction reflections were found to correspond to the Brag reflection specific to silver nanoparticles (27.832°, 32.253°, 46.261°, 54.834°, 57.501°, 67.461°, 76.748°). Miller indices (h-k-l) were calculated for each of these Brag reflections and accordingly values of (110), (111), (200), (231), (220), (311) and (222) were obtained for the synthesised silver nanoparticle (Fig. 3). These values are related to the face-centred cubic (fcc) lattice structure of the silver nanoparticle and are in accordance with the Joint Committee on Powder Diffraction Standards database (JCPDS No: 04–0783) (Mistry et al. 2021; Awad et al. 2022; Abu-Tahon et al. 2024). The state of this face-centered cubic (fcc) lattice structure is also related to the size of the synthesized nanoparticle. Using the Debye–Scherrer formula, the average crystallite size of the synthesized AgNPs was calculated to be 18.9 nm. In this study, the sharpest and strongest peak value of the fracture, expressed as “111” in the Miller index, has also been determined as another important indicator of the fcc structure. These observations in synthesized AgNPs have also been reported by researchers in similar mycosynthesis studies (Al-Soub et al. 2022; Hashem et al. 2022).

### SEM, TEM, zeta potential and EDX

**Fig. 4:**
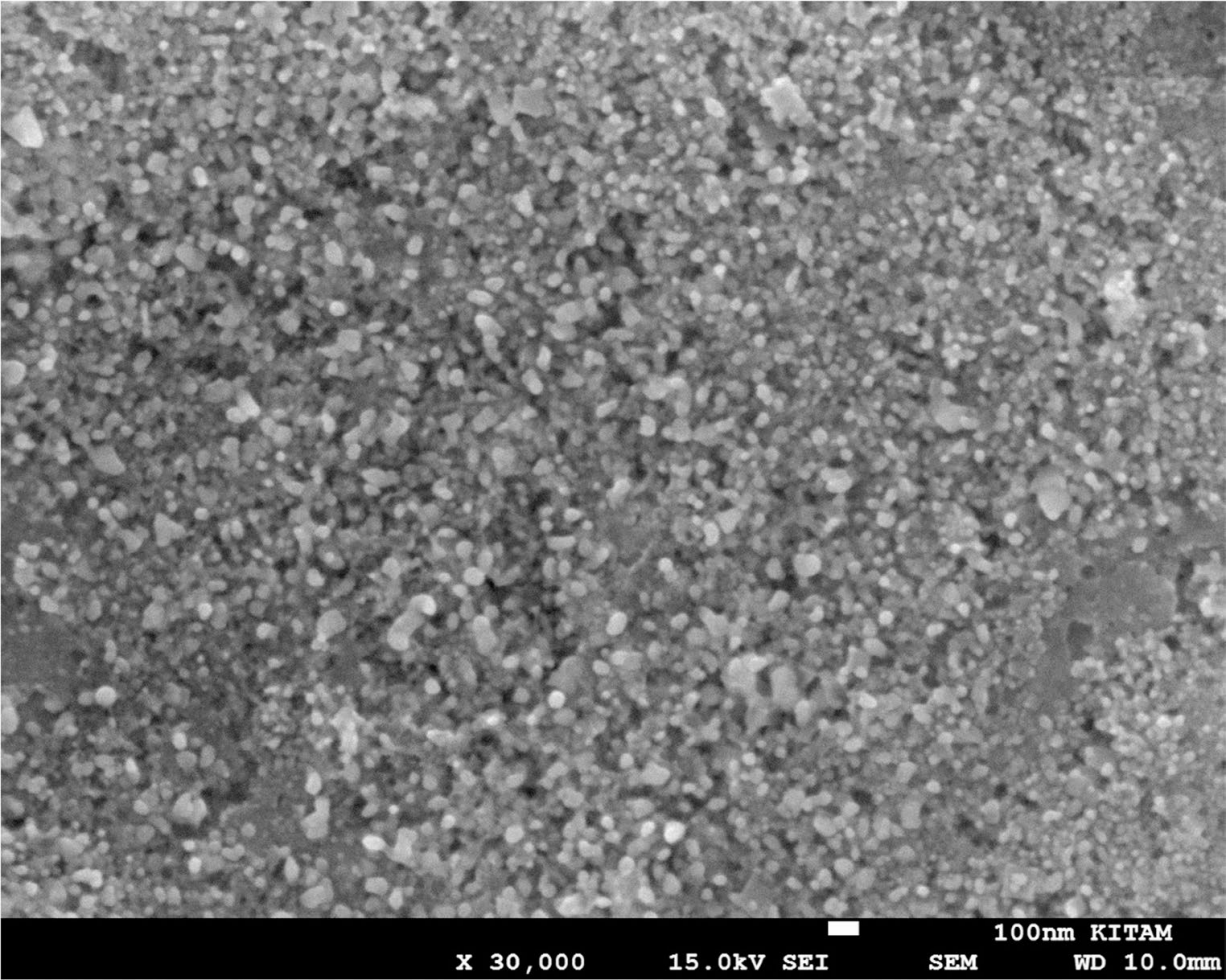
SEM image of AgNPs

**Fig. 5:**
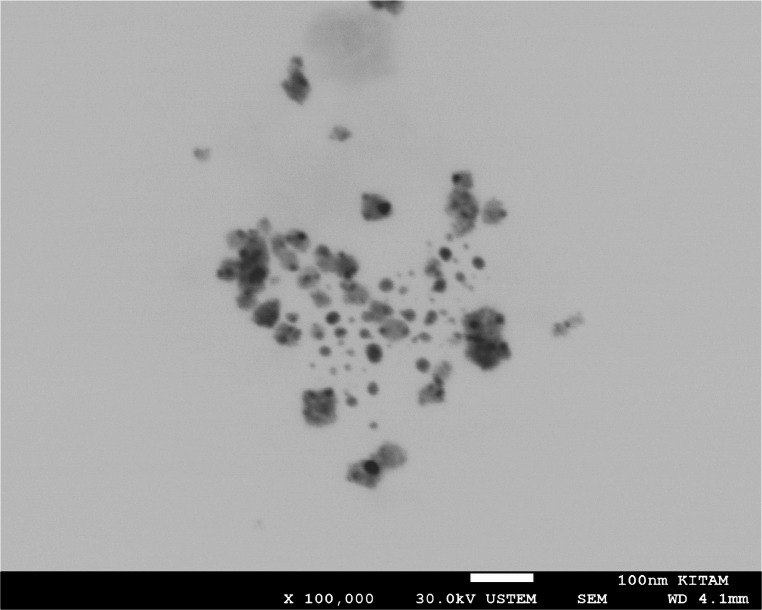
TEM image of AgNPs

**Fig. 6:**
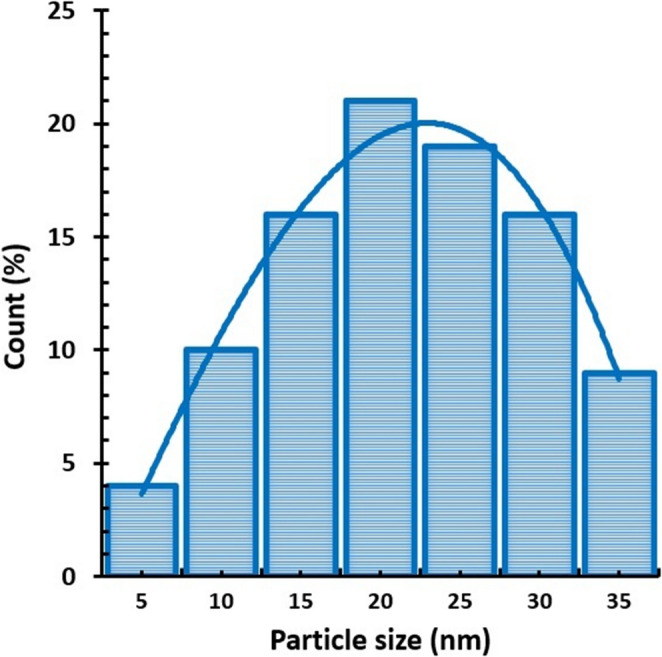
Particle diameter plot of AgNPs

**Fig. 7:**
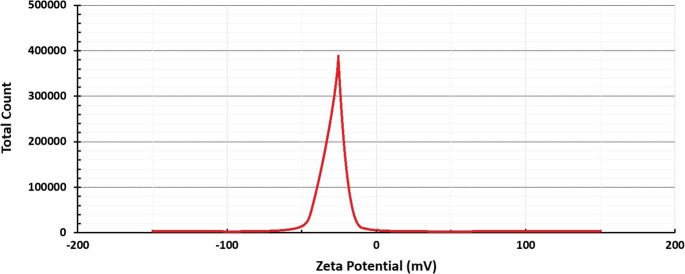
Zeta potential spectra of AgNPs

**Fig. 8:**
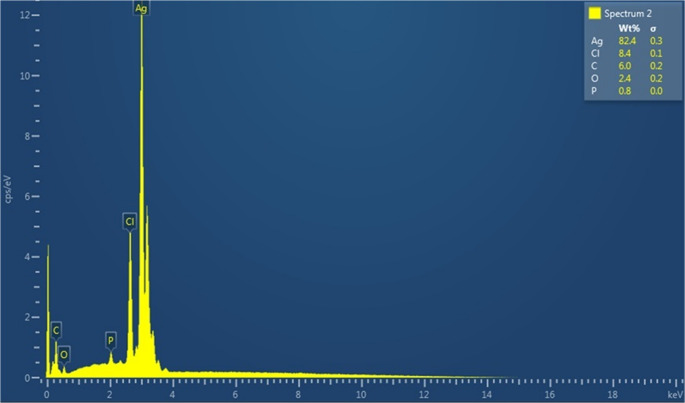
EDX spectrum of AgNPs

SEM images at X30 K magnification were used to determine the overall distribution and morphological structure of the synthesised silver nanoparticle, according to which it was determined that the nanoparticle has a spherical structure and exhibits a mixed cluster. The morphologically clearer shape, diameter and distribution of AgNPs were determined by TEM imaging analysis (using a TEM grid made of carbon-coated copper with 200 pores) at X100 K magnification, according to which it was found to exhibit a spherical structure and a slightly dispersed cluster structure with a diameter of 10–40 nm (Figs. 4 and 5). Similar morphological conditions were reported in SEM and TEM images of silver nanoparticles synthesised using similar fungi (*Aspergillus* spp.) (El-Bendary et al. 2021; Awad et al. 2022; Chi et al. 2022). As a result of processing the TEM image of AgNP in Image J programme, the diameters of AgNP can be calculated precisely and in this study, the diameters of AgNPs with spherical structure were determined to be between 5 and 35 nm (Fig. 6) (Baharara et al. 2014; Kumar et al. 2018b). As a result of Zeta potential analysis, −27.1 value was obtained and this negatively charged value indicates the stability of the synthesised silver nanoparticle (Fig. 7). Thanks to this negative charge, it is reported that silver nanoparticles have electrostatic repulsion power and with the effect of this power, both the clustering is at minimum level and they exhibit high dispersion and stability in the synthesis products developed in nano size (Macovei et al. 2022; Das et al. 2024). Energy dispersive X-ray spectroscopy (EDX) analysis was performed to determine the elements and their ratios on the synthesised silver nanoparticle and as a result, 82.4% silver (Ag), 8.4% Clor (Cl), 6.0% Carbon (C), 2.4% Oxygen (O) and 0.8% Phosphorus (P) elements were determined (Fig. 8). A specific feature of the AgNPs observed in the EDX spectrum is that the peak absorbance value of the silver element occurs exactly 3 keV above the graph and this is due to the surface plasmon resonance of AgNPs (Othman et al. 2021; Ali et al. 2024).

### Characterisation analyses of the fungal strain used in the mycosynthesis of the AgNPs

**Fig. 9:**
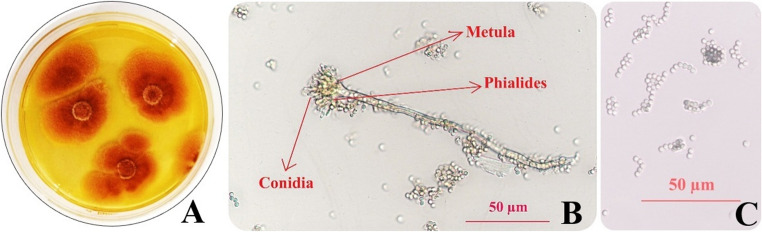
Morphological image of *Aspergillus floccosus* fungus, Macroscopic (**A**), Microscopic: conidia, metula and phialid structures (**B**), Microscopic: conidial image (**C**)

The growth of the fungus isolated from the soil and purified for use in AgNPs synthesis was monitored daily on PDA medium. The mycelium in the PDA culture medium was initially white in colour, but over time it was observed to turn orange-light brown. Mycelium growth reached a diameter of 30–40 mm after 5 days at 28 °C in the Petri dish (Fig. 9). When the micro-morphology of this fungus was evaluated under a microscope, descriptive morphometric data were obtained regarding the structures of the conidiophore, vesicle, phialide, and conidium. Conidiophores are single-veined, simple, with straight walls, sometimes curved, 321.75–1238.51 μm long, vesicles are spherical, some hemispherical, 30.5–58.7 × 41.2–61.6 μm in size, and phialides are bulbous, 6.5–12.7 × 3.1–3.8 μm in size. The conidia are spherical, some are semi-spherical and rough in structure, measuring 3.8–4.9 × 4.0–5.5 μm (Fig. 9). Based on the morphological and morphometric data obtained, it has been concluded with a high degree of probability that this fungus is *Aspergillus floccosus*. Similarly, in another study where the morphological characterisation of the *A. floccosus* isolate was performed (Pangging et al. 2022), the micro-morphological structure of the *A. floccosus* isolate isolated from soil was examined under a microscope. The conidial portion is long and dense, columnar in shape, Ø 45–95 μm in size, the conidiophores are double-rowed, 150–375 × 4.5–5.2 μm in size, the vesicles are spherical, Ø 12–16 μm in size, the metulae are tightly packed, 5.5–8.5. 8 × 1.8–2.1 μm in size, the phialides are 4.6–6.5 × 1.8–2.1 μm in size, and the conidia are spherical, elliptical, Ø 2.0–2.6 μm in size.

**Fig. 10:**
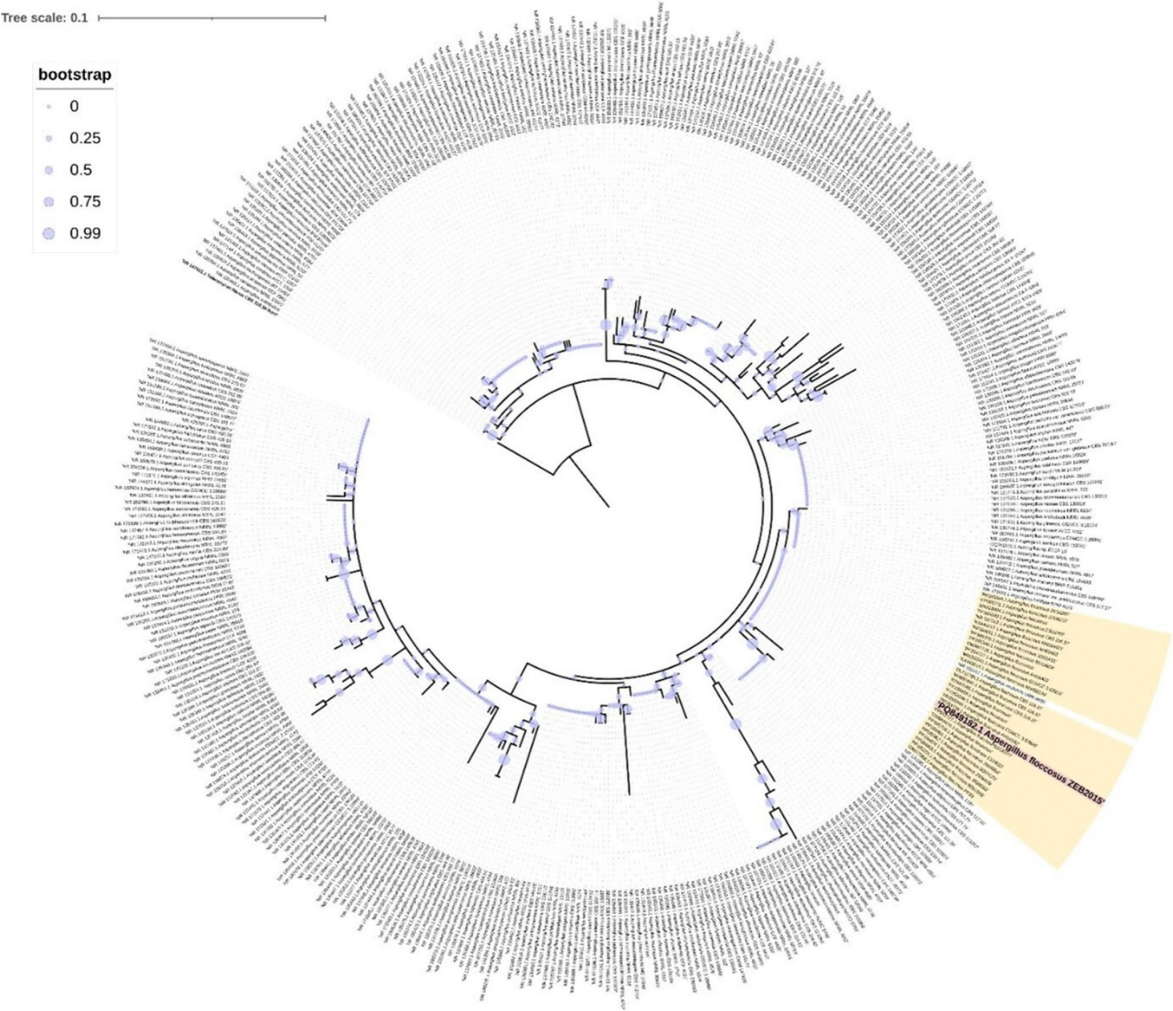
Phylogenetic tree of the fungus *Aspergillus floccosus.* *The ML tree obtained based on the phylogenetic analysis of the ITS sequence data of the isolated *Aspergillus floccosus* fungus (Ac. No. PQ849182.1) for this study. The numbers at the nodes indicate bootstrap values (≥ 50%) obtained from 1000 repetitions. The number of substitutions per nucleotide is indicated on the branches. The clade formed by *Aspergillus floccosus* species is shown in yellow, while the study isolate is indicated in bold and dark colour

The ITS gene region is considered the most important gene region in the molecular identification of fungi. For this study, the similarity of *Aspergillus* sp. isolated from soil to all *A. floccosus* species in the NCBI database, including those from South Korea (Ac. No. MN518404.1), France (Ac. No. PP851727.1), Cuba (Ac. No. OW984474.1) and China (Ac. No. KX443219.1), was determined to be between 99.82% and 99.45% based on ITS data. A phylogenetic tree was constructed using Clustal X version 2.1 (Thompson et al. 1997) to align all *Aspergillus* species samples obtained from GenBank (approximately 385 samples) with the ITS reference sequences of *A. floccosus* obtained from different countries (approximately 28 samples). For this purpose, manual edits were first made using Bioedit version 7.2.6.0 (Hall 1999), followed by the generation of “ML” phylogenies using MEGA version X. Subsequently, the Interactive Tree of Life (iTOL) server (https://itol.embl.de/) was used to visualise the phylogenetic trees in greater detail (Fig. 10). Phylogenetic analysis conducted using all *Aspergillus* sp. type specimens found in the GenBank database revealed that the *A. floccosus* ZEB2015 strain (accession number PQ849182.1) isolated for this study belongs to the same clade as similar species in the phylogenetic tree. A pure strain of *A. floccosus* was obtained as a result of the molecular identification of the fungal species isolated for this study. In one of the similar molecular diagnostic procedures performed considering the ITS gene region, the researchers preferred the same type of primer pair used in this study and molecularly identified the fungal isolate coded as MD1 as *Aspergillus terreus* (Ayad et al. 2024). Using the same type of ITS region primers, Hayat et al. (2023) identified the isolate isolated from soil and coded as ‘k1’ as *Penicillium notatum* and performed phylogenetic analyses using the MEGA 4.0 programme. Similarly, Khan et al. (2019) identified different fungal species in different media (SDA, PDA, and MEA) based on colony morphology and the ITS gene region as M1 (*Aspergillus niger* DGR1), M3 (*Aspergillus fumigatus* Ai2), M6 (*Aspergillus terreus* 1), and M7 (*Aspergillus flavus*).

### Characterisation analyses of the pathogenic fungal strain and evaluation of the pathogenicity test

**Fig. 11:**
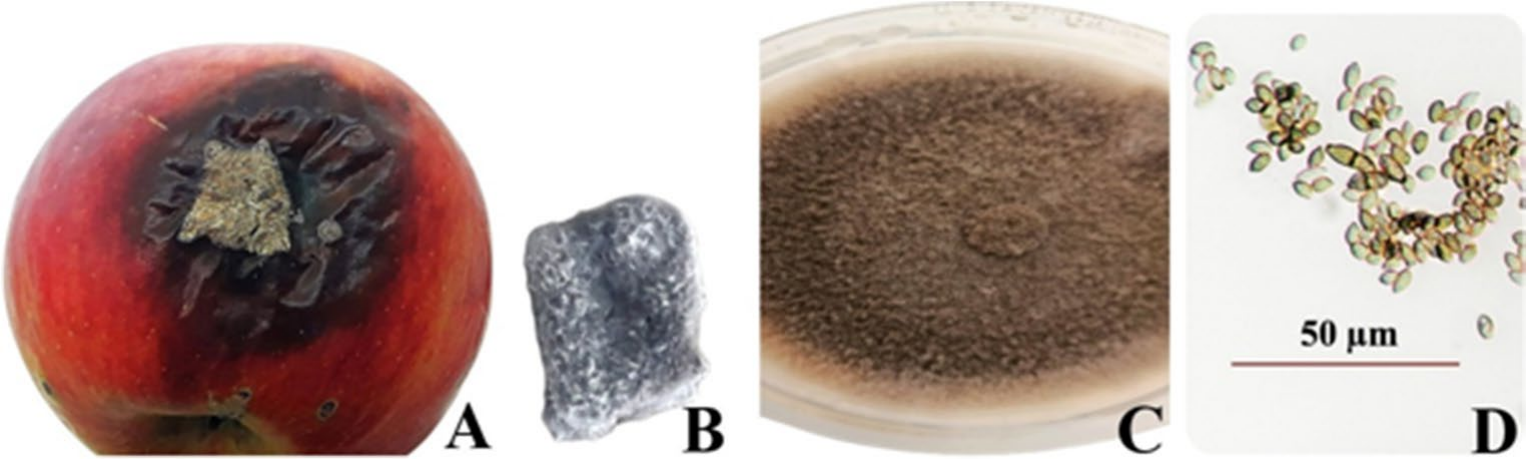
Morphological image of *Phoma herbarum* fungus, Apple fruit infected by apple rot disease (**A**), Fungal culture isolated from infected fruit (**B**), Macroscopic (**C**), Microscopic: conidial image (**D**)

The development of the fungus exhibiting symptoms of apple rot disease was observed day by day in PDA culture medium. It has been observed that the fungal culture developing on the PDA medium initially consists of white-light grey cottony mycelium, which later transforms into dense aerial mycelium and takes on a darker, greyish appearance. These conidia were glabrous and solitary, appearing subglobose with indistinct uniostiolate features, averaging 120–275 μm in diameter. Approximately 200 conidia were measured and their dimensions were recorded as 5–8.5 μm in length and 2.0–3.95 μm in width. These measurements are similar to the standard descriptions of the *Phoma herbarum* culture reported as 4.5–6.5 × 2–3 μm (Chen et al. 2015). This similarity ratio reinforces the validity of the morphological identification and characterisation of the pathogenic fungus isolated in this study as *P. herbarum.* (Fig. 11).

**Fig. 12:**
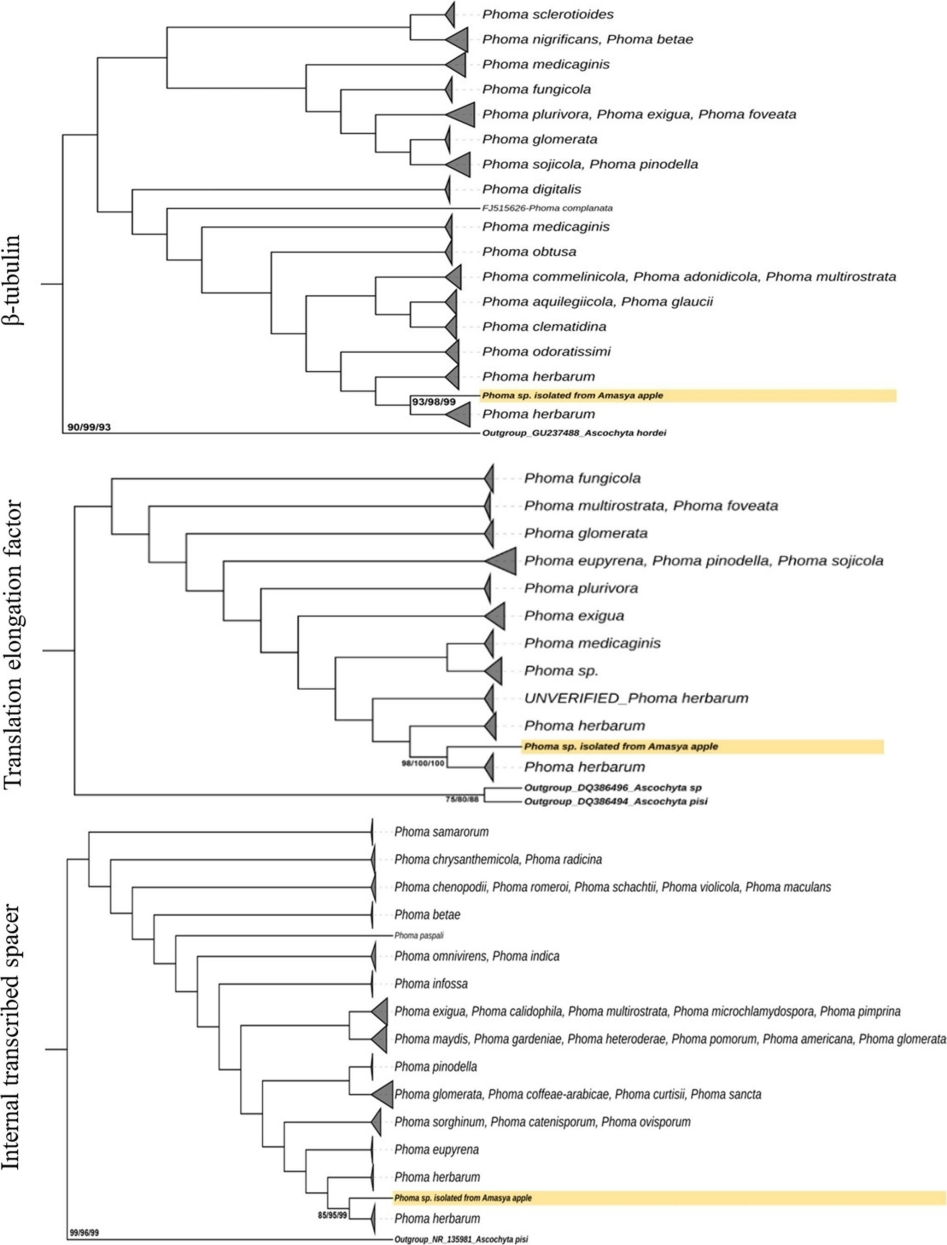
Phylogenetic tree of the fungus *Phoma herbarum.* *The fungal sample isolated as the causative agent of disease in apple fruit (Amasya cultivar) shows β-tubulin, translation elongation factor 1-α, and internal transcription spacers (Consensus phylogenetic trees obtained using NJ/ML/MP models, % Similarity). The numbers at the nodes indicate bootstrap values (*n* = 1000 repetitions), and the results of the NJ/ML/MP analyses are consistent with each other. β-tubulin (GU237488.1_*Ascochyta hordei*), translation elongation factor 1-α (DQ386494.1_*Ascochyta pisi* and DQ386496.1_*Ascochyta* sp.) and internal transcription spacer (NR135981_*Ascochyta pisi*) gene regions are shown in bold as outgroups (Aveskamp et al. 2009). In addition to the study, the β-tubulin, translation elongation factor 1-α, and internal transcription spacer sequences obtained from NCBI were combined to reconstruct the maximum likelihood (ML) tree (visualised using iTOL). In the resulting phylogenetic tree, Amasya *Phoma* sp. samples are marked with thick, dark and yellow stripes

Morphologically, *P. herbarum* can be distinguished based on its characteristics; however, given the complexities and overlaps within the *Phoma* genus, it is known that molecular methods such as analysis of the ITS, tef1 and tub2 gene regions are crucial for accurate identification. In their study on the phylogeny of *Phoma* taxa, Aveskamp et al. (2009) reconstructed and isolated the β-tubulin (107 DNA sequences), translation elongation factor 1-α (50 DNA sequences) and Internal Transcribed Spacer (379 DNA sequences) sequences using DNA data obtained from NCBI. It was found that more than 85% of the isolated samples belonged to *P. herbarum*. In this study, the phylogenetic analysis of the pathogenic fungus was performed using sequences obtained from three specific genetic loci: the β-tubulin gene, the Translation Elongation Factor 1-α gene, and the Internal Transcription Spacer (ITS) regions. The analysis provides a comprehensive insight into genetic diversity and phylogeny by visually displaying the relationships derived from sequence data (Fig. 12). The *Phoma* isolate obtained exhibited a clustering pattern within a specific phylogenetic clade, indicating its close genetic relationship with well-documented *P. herbarum* species. Research has revealed that this *Phoma* isolate clusters in a separate clade closely related to *P. herbarum*. Multilocus sequence analysis performed on this particular strain has also revealed that it belongs to the *P. herbarum* species (Fig. 12). Furthermore, the gene sequences obtained from this analysis have been further validated using the National Centre for Biotechnology Information (NCBI) Blast analysis. This verification process showed a significant degree of similarity with identity percentages ranging from 80% to 90% and confirmed with high reliability that this strain should be classified as *P. herbarum*. In this study, *Phoma* sp. species isolated from apples were identified based on the ITS region showed similarities (98.24–99.02%) with species from the Netherlands (MH855720), Spain (FN868459), Belgium (OW983234), Spain (LT592904) and Mexico (EU715683). When evaluated in terms of the translation elongation factor 1-α gene, *P. herbarum* isolates originating from the Netherlands (KF253168) and China (OK440236) showed over 90% homology. In terms of β-tubulin, it has shown homology with similar samples isolated from Germany (88%) (AY749025-AY749026-AY749027). The lower homology ratios observed in the translation elongation factor 1-α and β-tubulin genes, particularly in comparison to the ITS region, between the study isolate and other *P. herbarum* isolates have been interpreted as stemming from differences in sequence length and are thought to affect overall DNA sequence homology ratios. *P. herbarum* barcode sequences (GenBank accession numbers: ITS: PV390840, tef1: PV368857 and tub2: PV368856) were registered in NCBI GenBank.

According to the results of the pathogenicity test conducted on the selected and prepared apple fruits, it has been observed that the pathogenic fungus causes symptoms such as lesions and decay in apple fruits. No signs of infection were detected in the apples in the control group of the test. The percentage of infected inoculation points was calculated according to the relevant formula, and the disease incidence rate of the pathogenic fungus in apple fruits was determined to be 88.88%. At the same time, the results of the re-isolation test determined that the isolated pathogenic fungus was the same as the inoculated pathogenic fungus. In conclusion, the *P. herbarum* fungus, diagnosed at the morphological and molecular levels for this study, is a highly pathogenic organism that can cause serious rot symptoms in apple fruits. The fact that it causes such a high level of pathogenicity in apple fruits is also consistent with the study conducted by Stewart-Wade and Boland (2004). Additionally, similar diseases caused by *P. herbarum* have been observed in various crops; black spot disease in *Pisum sativum* (Li et al. 2011), leaf spot diseases in cherry palms (Kumla et al. 2016), in *Camellia sinensis* (Thangaraj et al. 2018), and in lilies (Sun et al. 2020). *P. herbarum* produces a phytotoxic glycoprotein known as anthraquinone. This toxin is highly glycosylated and is responsible for triggering the characteristic symptoms of the disease (Quereshi et al. 2011). Therefore, *P. herbarum* is known to have broad pathogenic capabilities on different plant species.

### Evaluation of the fungicidal nano-bioassay

**Fig. 13:**
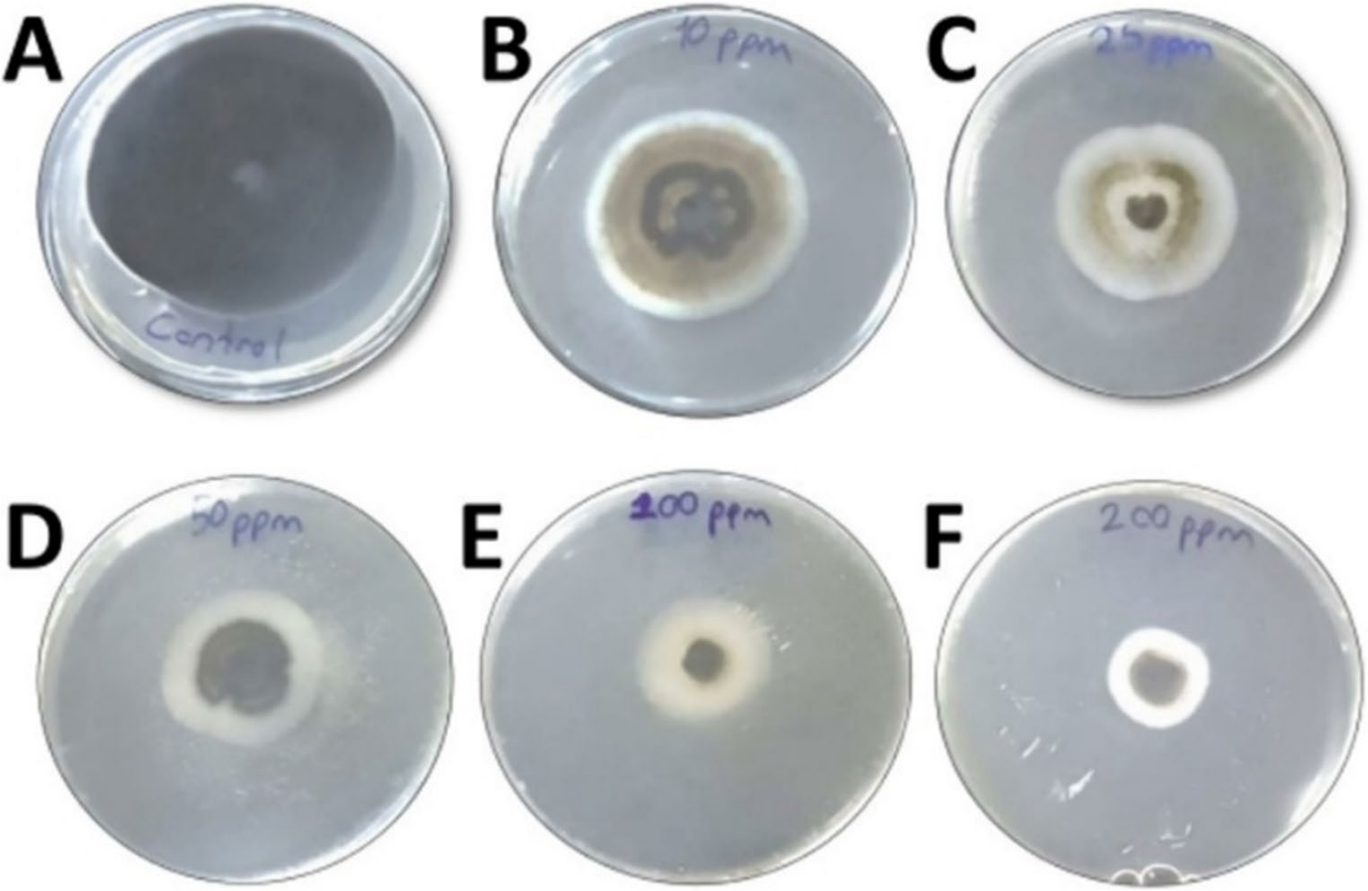
In vitro fungicidal nano-bioassay test results observed in petri dishes. *AgNP-0: Control-0 mg L⁻¹ (**A**), AgNP-10: 10 mg L⁻¹ (**B**), AgNP-25: 25 mg L⁻¹ (**C**), AgNP-50: 50 mg L⁻¹ (**D**), AgNP-100: 100 mg L⁻¹ (**E**), AgNP-200: 200 mg L⁻¹ (**F**)

The efficacy of AgNPs against *P. herbarum in vitro* tests was evaluated based on mycelial growth (mm) and the resulting inhibition rates (%). The application of AgNPs at different concentrations (AgNP-0, AgNP-10, AgNP-25, AgNP-50, AgNP-100 ve AgNP-200) is statistically significant in terms of mycelial growth (*p* ≤ 0.05). As a result, inhibition rates (%) were determined at different values. It was observed that as the applied AgNPs concentration increased, decreases in the mycelium diameter (mm) observed in the Petri dishes occurred, and as a result, increases in inhibition rates were observed (Fig. 13). Mycelial growth inhibition increased progressively with AgNP concentration, ranging from 18.2% at 10 mg L⁻¹ to 81.5% at 200 mg L⁻¹ (Table 1). The control group was accepted as the criterion, and inhibition percentages were calculated according to mycelium diameters. Accordingly, the lowest inhibition percentage was calculated as 18.2% with the application of AgNP-10 (10 mg L⁻¹), while the highest inhibition percentage was calculated as 81.5% with the application of AgNP-200 (200 mg L⁻¹) (Table 1). According to these results, under in vitro, the highest fungicidal activity against the pathogenic fungus (*P. herbarum*) was recorded at the highest AgNP concentration (AgNP-200).

**Table 1:** Effects of AgNPs application at different concentrations on pathogenic mycelial growth (mm) and Inhibition (%) under in vitro conditions

Concentrations of AgNPs (mg L⁻¹)	Diameter of Fungi (mm) ± SE	Inhibition rates (%)
AgNP-0 (Control)	70.93 ± 0.17^a^	---
AgNP-10	58.00 ± 0.62^b^	18.2
AgNP-25	48.00 ± 0.43^c^	32.3
AgNP-50	31.30 ± 0.08^d^	55.8
AgNP-100	24.60 ± 0.17^d^	65.3
AgNP-200	13.1 ± 0.24^e^	81.5

* Values shown with different letters within the same column indicate significant differences determined by Duncan’s multiple range test (*p* ≤ 0.05). These values have been statistically determined for each experiment in triplicate (± SE)

**Fig. 14:**
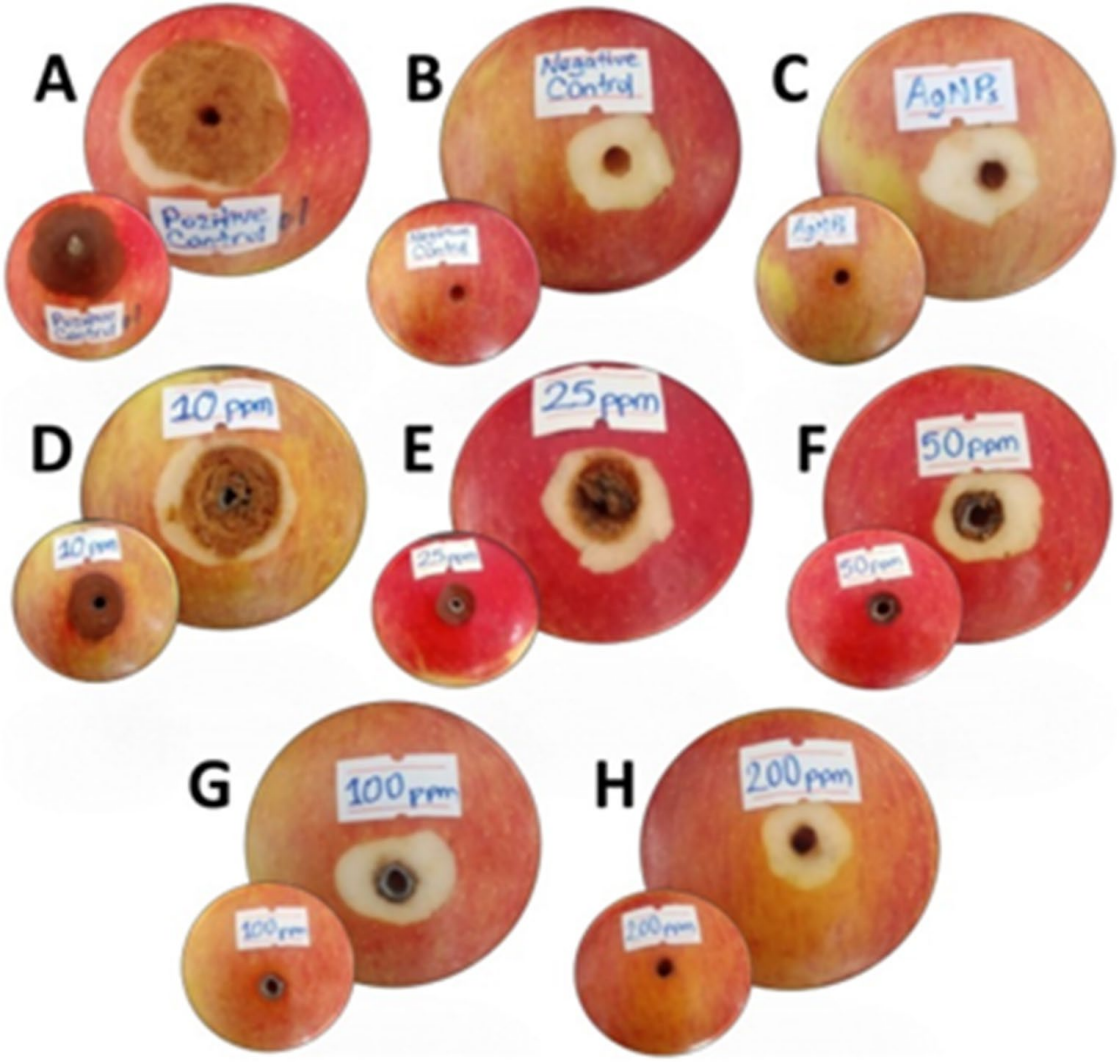
In vivo fungicidal nano-bioassay test results observed on apple fruits. *Positive Control (**A**), Negative Control (**B**), AgNP Effect Control (**C**), AgNP-10 + Fungi (**D**), AgNP-25 + Fungi (**E**), AgNP-50 + Fungi (**F**), AgNP-100 + Fungi (**G**), AgNP-200 + Fungi (**H**)

In vivo tests conducted on apple fruits have revealed differences in disease severity rates in apple fruits infected with pathogenic fungi, depending on the application model. (Fig. 14). In the assessment of the disease conditions that emerged, the rot diameter ratios associated with the rot formation observed in the apple fruits used in the application were first evaluated. When comparing the decay diameter ratios between applications, the results were found to be statistically significantly different (*p* ≤ 0.05). The highest observed fruit rot diameter ratio was recorded in the positive control application (Ø 51.00 ± 1.73 mm). The diameter ratios of observed fruit rot decreased in the following order: AgNP-10 (Ø 39.33 ± 0.57 mm), AgNP-25 (Ø 24.00 ± 1.00 mm), AgNP-50 (Ø 15.33 ± 1.52 mm) and AgNP-100 (Ø 10.00 ± 2.64 mm) applications. However, no signs of decay were observed in the fruits treated with the highest AgNPs concentration (AgNP-200), in the fruits treated with the negative control treatment, or in the fruits treated with the AgNPs effect control treatment (Table 2).

**Table 2:** The fungicidal efficacy of AgNP application at different concentrations against pathogenic fungi (*Phoma herbarum*) under in vivo conditions

Treatment	Decay diameter (mm) ± SE	DSI (%)	Disease reduction (%)
Positive control (Fungi)	51.00 ± 1.73^a^	92.00	---
Negative control (Water)	0.00 ± 0.00^f^	0	---
Effect control (AgNPs-200 mg L⁻¹)	0.00 ± 0.00^f^	0	---
AgNP-10 + Fungi	39.33 ± 0.57^b^	76.0	17.39
AgNP-25 + Fungi	24.00 ± 1.00^c^	53.3	42.06
AgNP-50 + Fungi	15.33 ± 1.52^d^	45.0	51.08
AgNP-100 + Fungi	10.00 ± 2.64^e^	20	67.39
AgNP-200 + Fungi	00.00 ± 0.00^f^	0	100

* The calculated decay diameters are the average of five apple fruits for each treatment (five replicates were performed for each treatment). Data are presented as mean values ± standard error. Mean values within the same column indicated by the same letter are not significantly different at the *p* ≤ 0.05 level according to Duncan’s multiple range test

Secondly, in the assessment of the disease conditions that emerged, the disease severity index (DSI) observed in the apple fruits used in the application was evaluated. The highest disease severity index was calculated in the positive control application (92%), followed in descending order by the AgNP-10 (76%), AgNP-25 (53.3%), AgNP-50 (45%) and AgNP-100 (20%) applications. The disease severity index was calculated as 0% in the highest AgNPs concentration application (AgNP-200), the negative control application, and the AgNP effect control applications (Table 2). Finally, in this in vivo study, disease reduction conditions were calculated and presented in tabular form. According to the results obtained, the highest disease reduction rate (highest fungicidal activity) was calculated and recorded as 100% in the application with the highest AgNPs concentration (AgNP-200). The disease reduction power decreased in direct proportion to the amount of concentration applied, and this ratio was calculated as 17.39% in the application with the lowest AgNPs concentration (AgNP-10) (Table 2).

Similarly to this study, researchers have developed AgNPs synthesis processes using fungi of the genus *Aspergillus*. At the end of these processes, nanoparticles with different properties and sizes were obtained, and their fungicidal activities were evaluated using these nano products. Roy et al. (2013) investigated the effect of AgNPs (20–40 nm) synthesised using the fungus *A. foetidus* (MTCC8876) on the growth of fungal mycelium under in vitro conditions. In the study, which utilised the plant pathogens *A. niger*,* A. foetidus*,* A. flavus*,* Fusarium oxysporum*,* A. oryzae*, and *A. parasiticus*, it was determined that the mycelium growth inhibition zone varied between 1.5 and 2 cm on PDA medium, depending on the fungal species. At the end of this study, in which *A. floccosus* fungus was preferred, it was determined that the synthesized AgNPs were in the range of 5–35 nm. Although similar fungi were used for synthesis, it is reported that some reasons for obtaining different nanoparticle sizes at the end of synthesis are directly related to the fungal enzymatic profile, metabolite production capacity, and reduction-stability mechanisms (Ali et al. 2025). The influence of the biochemical properties of isolates belonging to different species of the same genus on nanoparticle size more accurately explains why synthesized AgNPs exhibit different fungicidal activities. Consequently, it can be assumed that different fungicidal activities may be observed due to different AgNPs sizes for different fungi. Elgorban et al. (2016) investigated the effectiveness of AgNPs (5–30 nm) synthesized using *A. versicolor* isolates against *Sclerotinia sclerotiorum*, the causative agent of white rot in strawberries, and *Botrytis cinerea*, the causative agent of gray rot. Researchers have determined that the synthesized AgNPs exhibit a potent fungicidal effect against both pathogens, and that this effect is dependent on nanoparticle size and dose.

In this study, the pathogen *P. herbarum* fungus is a new species with the potential to cause rot in apples, and therefore, no fungicidal activity tests at the nanotechnological level have been conducted against this fungus to date. However, the effects of AgNPs against fungal agents causing apple rot disease have been investigated in vitro and in vivo in different studies. Malandrakis et al. (2020) determined in their study that a 100 mg L^− 1^ AgNPs (< 100 nm) concentration reduced the disease severity of the apple rot pathogen *Monilinia fructicola* by 6.75–16.67%. When the same dose of AgNPs was applied against *P. herbarum*, the causative agent of rot, the disease rate decreased by 67.39%. Talie et al. (2020) investigated the fungicidal effect of AgNPs (80 to 100 nm) at different concentrations on the pathogenic fungi *Penicillium chrysogenum*,* A. niger*, and *Alternaria alternata*, which are the causative agents of apple rot. The results showed that the highest dose of 20 mg L^− 1^ inhibited the mycelial growth of the pathogens *P. chrysogenum*,* A. niger*, and *A. alternata* by 83.21%, 77.32%, and 69.10%, respectively. Madbouly (2021) investigated the effect of AgNPs (20–170.6 nm) at different concentrations (25, 50, 100, and 200 mg L^− 1^) against the post-harvest apple brown rot pathogen *M. fructigena* under in vitro and in vivo conditions. According to his findings, AgNPs inhibited the mycelial growth of *M. fructigena in vitro* by 33.1–96.1%, depending on the concentration. Under in vivo conditions, they determined that the diameter of the rot in the fruit decreased by 56.6%, 40.7%, 64.9%, and 68.9%, respectively, depending on the different concentrations of AgNPs. Although the average size of AgNPs synthesized using *A. floccosus* isolates was 20 nm, they inhibited *P. herbarum* mycelial growth to a lesser extent (18.2–81.5%) in vitro. However, it is thought that the differences in fungicidal activity observed in the fungicidal tests of these three studies may also be related to the sizes of the nanoparticles applied or the characteristic structure of the AgNPs.

Nanoparticle size is quite important in explaining the fungicidal effect of AgNPs, and it has been reported that AgNPs with particles smaller than 20 nm are more effective against microorganisms because they can penetrate cells more easily (Duman et al. 2024). Of course, while the fungicidal effect of AgNPs against fungi varies depending on the size of the nanoparticles, ultimately all AgNPs exert significant pressure on fungi. When this effect is examined at the cellular level, AgNPs disrupt the fungal cell membrane, causing cellular components to leak out of the cell (Stoimenov et al. 2002). Additionally, AgNPs disrupt the fungal respiratory chain at the cellular level and cause cell death by inhibiting fungal cell division (Khalil et al. 2019). Additionally, AgNPs inhibit cellular enzymes by releasing Ag⁺ ions, can affect the electron transport chain, cause DNA damage, and severely disrupt cellular processes (Jian et al. 2022; Almeida et al. 2024). The inevitable fate of fungi whose cell structure has been damaged by AgNPs and can no longer perform their functions is extinction.

Synthetically produced AgNPs, such as those synthesized from microorganisms, have the ability to inhibit mycelial growth in plant pathogenic fungi. Karimi and Sadeghi (2019) investigated the effects of commercially produced AgNPs (50, 100, and 150 mg L^− 1^) and two different synthetic fungicides on pathogens (*Alternaria alternata*,* Alternaria citri*, and *Penicillium digitatum*) isolated from citrus samples under in vitro conditions. The results of the study showed that AgNPs possessed stronger antifungal activity against the isolated pathogens compared to fungicides. However, the use of biological extracts such as fungi in AgNPs synthesis is both more economical and less toxic compared to synthetic AgNPs synthesis (Guilger-Casagrande and Lima 2019). Because the goal here is not only to develop nano products that are effective against pathogenic fungi, but also to create an alternative nano pesticide with minimal toxic effects on the environment. Therefore, researchers indicate that the use of nano products synthesized via green synthesis is safer in AgNPs studies against pathogenic fungi in agriculture (Malik et al. 2024).

### Evaluation of the insecticidal nano-bioassay

Results showed that the effectiveness of AgNPs against pests is directly related to the dose amount and exposure time. Increasing the dose amount and exposure time increases mortality rates. According to the results of the probit analysis, as exposure time increases, the dose required for a lethal effect decreases (Table 3). Similarly, as the dose increases, the exposure time required for a lethal effect decreases (Table 4). When the efficacies of both nano-biological tests were compared over the exposure times to nanoparticles, it was determined that the dose required to kill adults (LC50: 48.926 mg L⁻¹ for lab. app. and LC50: 68.424 mg L⁻¹ for orc. app.) was higher than the dose required to kill nymphs (LC50: 32.273 mg L⁻¹ for lab. app. and LC50: 52.073 mg L⁻¹ for orc. app.) (Table 3). As a result of the evaluation of the effectiveness of nano-bioassay applications over the dose amounts applied against pests, it was determined that the exposure times required to kill adults (51.167 h for LT50: lab. app. and 58.438 h for LT50: orc. app.) at the highest dose rate (150 mg L⁻¹) were higher than the exposure times required to kill nymphs (42.024 h for LT50: lab. app. and 45.125 h for LT50: orc. app.) (Table 4).

**Table 3:** LC50 and LC95 values obtained after spraying mycosynthesised AgNPs on nymphs and adults of *Aphis Pomi* de Geer

		*Laboratory Application (Lab. app.)*					*Apple Orchard Application (Orc. app.)*				
	Time (hour)	LC50 (mg L⁻¹)	LC95 (mg L⁻¹)	Slope ± SE	X^2^ (df)	p	LC50 (mg L⁻¹)	LC95 (mg L⁻¹)	Slope ± SE	X^2^ (df)	p
***Fourth instar nymph***	**24**	215.944	536.469	0.005 ± 0.001	5.611 (48)	0.999	210.968	486.263	0.006 ± 0.001	7.955 (48)	0.999
	**48**	119.497	335.867	0.008 ± 0.001	10.062 (48)	0.999	129.994	325.121	0.008 ± 0.001	9.501 (48)	0.999
	**72**	71.798	214.559	0.010 ± 0.001	11.727 (48)	0.999	87.171	267.077	0.009 ± 0.001	13.998 (48)	0.999
	**96**	32.273	158.363	0.012 ± 0.002	15.889 (48)	0.999	52.073	205.211	0.011 ± 0.001	14.631 (48)	0.999
***Adult***	**24**	224.103	554.991	0.004 ± 0.002	6.840 (48)	0.999	257.848	592.823	0.005 ± 0.002	7.683 (48)	0.999
	**48**	111.174	316.832	0.006 ± 0.001	11.834 (48)	0.999	169.851	441.368	0.006 ± 0.001	9.405 (48)	0.999
	**72**	73.954	217.064	0.008 ± 0.001	12.323 (48)	0.999	111.481	342.549	0.007 ± 0.001	7.390 (48)	0.999
	**96**	48.926	177.332	0.010 ± 0.001	23.792 (48)	0.999	68.424	256.902	0.009 ± 0.001	7.322 (48)	0.999

* LC50: Lethal concentration killing 50% of exposed organisms; **LC95**: Lethal concentration killing 95% of exposed organisms; **χ2** : Chi-square test; **p**: Significance of χ2

* Lab. app.: Laboratory application; **Orc. app.**: Orchard application

**Table 4:** LT50 and LT95 values obtained after spraying mycosynthesised AgNPs on nymphs and adults of *Aphis Pomi* de Geer

		*Laboratory Application (Lab. app.)*					*Apple Orchard Application (Orc. app.)*				
	Conct. (mg L⁻¹)	LT50 (hour)	LT95 (hour)	Slope ± SE	X^2^ (df)	p	LT50 (hour)	LT95 (hour)	Slope ± SE	X^2^ (df)	p
***Fourth instar nymph***	**30**	100.249	214.702	0.014 ± 0.003	14.413 (38)	0.999	116.301	242.750	0.013 ± 0.003	12.268 (38)	0.999
	**60**	79.256	189.401	0.015 ± 0.002	5.312 (38)	0.999	92.402	215.833	0.013 ± 0.003	5.346 (38)	0.999
	**90**	52.192	144.122	0.018 ± 0.003	4.629 (38)	0.999	62.176	167.241	0.016 ± 0.002	7.905 (38)	0.999
	**120**	47.552	134.587	0.019 ± 0.003	4.937 (38)	0.999	53.751	153.621	0.016 ± 0.002	9.196 (38)	0.999
	**150**	42.024	107.319	0.025 ± 0.003	7.048 (38)	0.999	45.125	127.227	0.020 ± 0.003	6.428 (38)	0.999
***Adult***	**30**	119.226	256.549	0.012 ± 0.003	12.585 (38)	0.999	123.006	249.496	0.013 ± 0.003	4.093 (38)	0.999
	**60**	81.571	191.478	0.015 ± 0.003	5.516 (38)	0.998	98.878	237.097	0.012 ± 0.003	3.271 (38)	0.999
	**90**	68.086	176.509	0.015 ± 0.002	8.288 (38)	0.999	81.536	221.903	0.012 ± 0.002	4.318 (38)	0.999
	**120**	58.475	148.553	0.018 ± 0.003	18.454 (38)	0.997	66.422	169.307	0.016 ± 0.002	8.179 (38)	0.999
	**150**	51.167	122.152	0.023 ± 0.003	4.980 (38)	0.999	58.438	149.813	0.018 ± 0.003	6.393 (38)	0.999

* **LT50**: Lethal time killing 50% of exposed organisms; **LT95**: Lethal time killing 95% of exposed organisms; **χ2** : Chi-square test; **p**: Significance of χ2

* Conct.: Concentration; **Lab. app.**: Laboratory application; **Orc. app.**: Orchard application

Many fungi with different properties are preferred for fungus-based nano-synthesis reactions in the green synthesis of AgNPs. The nano-products obtained at the end of the synthesis process using these extracts are used in determining different biological activities (Fouda et al. 2022; Abd Elghaffar et al. 2024; Mehmood et al. 2024). However, no AgNPs synthesis study has been found in the literature using the soil-derived fungus *A. floccosus* and this study is the first AgNPs synthesis and AgNPs-induced insecticidal activity study using this fungal species. In AgNPs studies conducted with *Aspergillus* spp. fungi, insecticidal efficacy evaluations have addressed different application methods against different pests (Soni and Prakash 2013; Awad et al. 2022). Soni and Prakash (2013) applied AgNPs synthesised (20–70 nm) from *A. niger* fungus at six different doses (2, 4, 6, 8, 10 and 12 mg L⁻¹) against *Cx. quinquefasciatus*,* Ae. aegypti* and *An. stephensi* mosquitoes and observed that mortality rates increased as the dose amount and application time increased. Awad et al. (2022) applied AgNPs (2–13 nm) obtained from *A. niger* fungus at six different application doses (5, 10, 15, 20, 25 and 30 mg L⁻¹) against larval instars (I, II, III and IV instars) and pupae of *A. aegypti* in the laboratory. According to the data obtained at the end of twenty-four hours, the mortality values in LC90 for larval instars (I, II, III and IV instars) and pupae were determined as 22.9, 22.4, 24.6, 27.1, 37.6 and 41.4 mg L⁻¹, respectively. In these two studies conducted on AgNPs synthesized from *Aspergillus* fungi, the nanoparticle size is generally between 2 and 70 nm. In this study, which examined AgNPs synthesized using the *A. floccosus* fungus, nanoparticle sizes ranged from 5 to 35 nm (approximately 19 nm). The particle sizes of AgNPs synthesized using extracts obtained from different *Aspergillus* species were found to range from 5 nm to 185 nm (*A. fumigatus*: 5–95 nm; *A. clavatus*: 25–145 nm; *A. niger*: 25–175 nm; *A. flavus*: 45–185 nm) (Zomorodian et al. 2016).

The high insecticidal activity of AgNPs obtained by green synthesis, even at low application doses, is believed to be primarily related to their nano size (1–100 nm) (Suresh et al. 2018; Awad et al. 2022). Thanks to their nano structure, AgNPs adhere to the insect’s cuticle and rapidly penetrate the body, affecting many physiological mechanisms (primarily the digestive and respiratory systems) (Rai et al. 2014; Jiang et al. 2015; Benelli 2016; Srinivasan et al. 2023). AgNPs entering the cells that play a role in the physiological mechanisms of the insect by using different systems (such as binding to different transmembrane structures in the cell membrane) can prevent or disrupt the functioning of cellular reactions (reactive oxygen species-ROS, enzyme synthesis mechanisms, DNA synthesis systems) that play an important role in cells (Kamaraj et al. 2017; Benelli 2018; Mao et al. 2018; Jin et al. 2020). Exposure to excessive levels of Ag^+^ ions reaching the cell disrupts the ROS mechanism, leading to destructive conditions that increase stress within the cell and cause serious damage (Zhu et al. 2017; Li et al. 2020). As a result of cellular damage, cells become dysfunctional in a short period of time and subsequently cellular destruction processes begin and finally cellular death is observed (Jiang et al. 2015; Suresh et al. 2018; Amjad et al. 2022). This cellular destruction and annihilation take place very quickly and the target insect dies within a very short time (Roesslein et al. 2013; Kamaraj et al. 2017; Benelli 2018).

The insecticidal effect of synthesized AgNPs is related not only to nanoparticle size, but also to the dose of the nano product applied, the duration of exposure to the nano product, and the biological stage of the insect. In this study, attention was drawn to these important factors and the importance of the results obtained on insecticidal activity was determined. In similar studies, it has been clearly stated by researchers that these important factors are crucial for AgNPs-based insecticidal studies. Shahid et al. (2022) applied synthesised AgNPs (7–10 nm) at different doses (200, 400, 600, 800, 1000 and 1200 mg L⁻¹) to both adults and nymphs (3rd instar) of *Bemisia tabaci* (Homoptera; Aleyrodidae). It was determined that mortality rates increased as the exposure time to AgNPs increased and the highest mortality rate occurred at the highest dose application (1200 mg L⁻¹ at the end of the fourth day; 77.77% for adults and 82.22% for nymphs). Hasoon et al. (2023) applied different doses (25, 50 and 100 mg L⁻¹) of AgNPs (85 nm) to two different biological stages (nymph and adult) of *Schizaphis graminum* (Rondani) (Hemiptera: Aphididae). As the dose and exposure time increased, the observed mortality rates also increased. At the end of the third day, the highest mortality rate was recorded in nymphs (57.13%) at the highest dose rate (100 mg L⁻¹), while the mortality rate in adults was 52.99%. AgNPs synthesised using *A. floccosus* fungal extract proved to be highly effective against both nymph and adult stages of the agricultural pest considered in this study in a short time even at very low dose values (Tables 3 and 4).

Exposing target pests to low doses of AgNPs causes them to die quickly; however, in nature, this situation may also pose a small risk factor for natural predators. Studies have shown that predatory insects are much less affected by very low doses of AgNPs used against target pests, because these organisms are stronger and more resistant. Therefore, it is recommended that insecticidal efficacy studies be conducted at low doses for these AgNPs (Benelli 2016; Kumar et al. 2016). Additionally, the green method used to synthesize AgNPs is an easier procedure than direct chemical synthesis and is less toxic (Choudhary et al. 2024). Furthermore, it is reported that it will not create a toxic effect on non-target organisms and will not create an adverse situation for the environment when used correctly, taking into account the specified dose amounts for the target pest group (Benelli et al. 2017; Kumar et al. 2022; Wang et al. 2024). In addition to all this, the fundamental advantage of green synthesis in AgNPs production is that it is both ecological and economical. This enables the use of environmentally friendly and biodegradable materials. Thanks to these nano products, which are considered a sustainable method, the likelihood of harming the environment and nature after nano pesticide applications is reduced (Hadjidemetriou and Kostarelos 2017; Santos et al. 2021).

## Conclusion

Intensive chemical control measures are taken every year against fungi and pests that cause significant quality and quantity losses in apple production. However, this situation reaches quite dangerous dimensions both in terms of causing residues in apples and causing resistance development in fungi and pests. This study, initiated to determine the feasibility of using AgNPs synthesized via green synthesis as a nanopesticide product against fungi and pests harmful to apple cultivation, has been successful. This nano-pesticide, which is a highly effective product that can be an alternative to chemical control, was highly effective against two harmful organisms (*P. herbarum* and *A. pomi*) at very low doses and in very short periods. This study on AgNP-based nanopesticides is expected to guide the development of innovative products for plant health through nanotechnological applications.

## Data Availability

No datasets were generated or analysed during the current study.
